# Nuclear receptor ERRα contributes to castration-resistant growth of prostate cancer via its regulation of intratumoral androgen biosynthesis

**DOI:** 10.7150/thno.35589

**Published:** 2020-03-04

**Authors:** Zhenyu Xu, Taiyang Ma, Jianfu Zhou, Weijie Gao, Youjia Li, Shan Yu, Yuliang Wang, Franky Leung Chan

**Affiliations:** 1 Precision Medicine Centre, Yijishan Affiliated Hospital of Wannan Medical College, Wuhu, Anhui Province, PR China; 2 School of Biomedical Sciences, Faculty of Medicine, the Chinese University of Hong Kong, Hong Kong, China; 3 Department of Urology, The Second Affiliated Hospital of Guangzhou University of Chinese Medicine, Guangzhou, China

**Keywords:** ERRα, nuclear receptor, AKR1C3, CYP11A1, intratumoral steroidogenesis, castration resistance, prostate cancer

## Abstract

Enhanced intratumoral androgen biosynthesis and persistent androgen receptor (AR) signaling are key factors responsible for the relapse growth of castration-resistant prostate cancer (CRPC). Residual intraprostatic androgens can be produced by *de novo* synthesis of androgens from cholesterol or conversion from adrenal androgens by steroidogenic enzymes expressed in prostate cancer cells via different steroidogenic pathways. However, the dysregulation of androgen biosynthetic enzymes in CRPC still remains poorly understood. This study aims to elucidate the role of the nuclear receptor, estrogen-related receptor alpha (ERRα, *ESRRA*), in the promotion of androgen biosynthesis in CRPC growth.

**Methods**: ERRα expression in CRPC patients was analyzed using Gene Expression Omnibus (GEO) datasets and validated in established CRPC xenograft model. The roles of ERRα in the promotion of castration-resistant growth were elucidated by overexpression and knockdown studies and the intratumoral androgen levels were measured by UPLC-MS/MS. The effect of suppression of ERRα activity in the potentiation of sensitivity to androgen-deprivation was determined using an ERRα inverse agonist.

**Results**: ERRα exhibited an increased expression in metastatic CRPC and CRPC xenograft model, could act to promote castration-resistant growth via direct transactivation of two key androgen synthesis enzymes CYP11A1 and AKR1C3, and hence enhance intraprostatic production of dihydrotestosterone (DHT) and activation of AR signaling in prostate cancer cells. Notably, inhibition of ERRα activity by an inverse agonist XCT790 could reduce the DHT production and suppress AR signaling in prostate cancer cells.

**Conclusion**: Our study reveals a new role of ERRα in the intratumoral androgen biosynthesis in CRPC via its transcriptional control of steroidogenic enzymes, and also provides a novel insight that targeting ERRα could be a potential androgen-deprivation strategy for the management of CRPC.

## Introduction

Androgen receptor (AR) signaling is the major pathway in prostate cancer and thus androgen- deprivation therapy still remains the current principal treatment option for locally advanced and metastatic prostate cancer. However, acquired resistance to therapy still invariably develops in patients and progress to therapy-resistance. Failure to androgen- deprivation or hormone therapy developed in advanced metastatic prostate cancer patients is the primary cause of mortality among patients and is still a significant clinical problem 1. Among multiple adaptive mechanisms in response to hormone therapy, persistent AR signaling is believed as one of the major factors contributing to recurrence of therapy- or castration-resistant prostate cancer (CRPC) 2, 3. Besides AR gene-related mechanisms (such as AR gene amplification and its overexpression, mutation, and splice variation), intratumoral androgen biosynthesis is considered as another key factor responsible for the persistent elevated tissue levels of androgens causing the reactivation of AR signaling in CRPC tissue despite low levels of circulating androgens upon the systemic androgen- deprivation therapy or castration 4. Based on this understanding, targeting the persistent AR signaling axis is still the current mainstay therapeutic option for the management of CRPC 5, 6. In fact, xenograft models of CRPC and CRPC tissues show increased expressions of multiple key steroidogenic enzymes involved in the biosynthesis of testosterone (T) and dihydrotestosterone (DHT) using several pathways, including the classical (canonical or front-door) pathway (by conversion of adrenal androgens dehydroepiandrosterone/DHEA→androstenediol→T→ DHT), alternative “*backdoor*” pathway (progesterone →→androstanediol→DHT or DHEA→→5α-Adione →DHT) or direct *de novo* synthesis from cholesterol 7-12. Based on this, it forms the pharmacological basis of inhibition of systemic or intratumoral biosynthesis of androgens by targeting the androgen synthesis enzymes (such as the CYP17A1 inhibitor abiraterone acetate) as a novel hormone or androgen-deprivation therapy for the management of metastatic CRPC 13, 14.

Estrogen-related receptor alpha (ERRα, NR3B1, *ESRRA*) is a constitutive active ligand-independent orphan nuclear receptor belonging to the nuclear receptor superfamily. ERRα shares significant structural similarities with the ligand-dependent estrogen receptors (ERα and ERβ) but does not bind estrogens 15. Studies in past decades characterize that ERRα, working with its co-regulators peroxisome proliferator-activated receptor γ coactivator-1 α and β (PGC-1α and PGC-1β), performs a central role in regulation of cellular energy metabolism and mitochondrial functions, and functions in many biological processes 16. Increased expression of ERRα and dysregulated function of ERRα-PGC-1 axis are demonstrated in different cancers and also associated in their unfavorable clinical outcomes 16, 17. We and others demonstrate that ERRα exhibits an up-regulation pattern in advanced prostate cancer 18, 19, and is implicated as a negative prognostic marker for prostate cancer 20. Our previous study shows that ERRα can function to promote the hypoxic growth adaptation of prostate cancer cells via its direct interaction with HIF-1α and enhancement of HIF-1 signaling 21. Study in an intra-osseous prostate cancer xenograft model shows that overexpression of ERRα can promote the *in vivo* growth of prostate cancer cells in bone via stimulation of bone cells and modulation of extracellular matrix in stroma 22. Our recent study shows that increased co-expression of ERRα and an oncogenic transcription factor ERG expressed by TMPRSS2:ERG fusion gene is closely associated with the Gleason scores and metastasis status in prostate cancer, and both ERRα and ERG can synergistically regulate each other at transcriptional level and form a reciprocal regulatory loop to promote the advanced growth of prostate cancer 18.

It is believed that up-regulation of steroidogenic enzymes involved in androgen biosynthesis in prostate cancer tissues is a main driving force for the CRPC. However, the mechanism or factors involved in the regulation of androgen biosynthetic enzymes still remains not well understood. The main goal of the present study was to determine the role of ERRα in the growth regulation of CRPC. Here, we showed that ERRα, which exhibited an up-regulation pattern in metastatic CRPC and also xenograft model of CRPC, could function to promote the resistance to androgen-deprivation in prostate cancer cells and also enhance the intratumoral androgen biosynthesis via its transcriptional regulation of some key androgen biosynthetic enzyme genes, including CYP11A1 and AKR1C3. Our results also implicate that ERRα could be a potential therapeutic target as its inhibition could suppress the activation of AR signaling in prostate cancer cells.

## Methods

### Cell lines and cell culture

Three human prostate cancer cell lines (LNCaP, VCaP and DU145; ATCC, Manassas, VA), one embryonic kidney cell line HEK293 and its SV40 large T- antigen-transformed subline HEK293FT (Invitrogen), and the viral packaging mouse cell line PA317 (ATCC) were used in this study. LNCaP cells were maintained in RPMI1640 supplemented with 4.5 g/liter glucose, 1 mM sodium pyruvate, 10 mM HEPES and 10% FBS; VCaP and PA317 cells in DMEM with 10% FBS; DU145 cells in MEM with 1 mM sodium pyruvate and 10% FBS; HEK293 and HEK293FT cells in DMEM with 10% FBS; with cells cultured with or without 50 U/ml penicillin- streptomycin mixture. The cells and their sublines were harvested when grown to 70-80% confluence for various analyses.

### Gene expression data analysis

The survival analysis of ERRα expression was performed in CRPC patients using an exome sequencing (RNA-seq) dataset (GSE35988; Grasso et al., 2012) from the Gene Expression Omnibus (GEO; https://www.ncbi.nlm.nih.gov/geo/) 23. The expression profiles of ERRα and two key steroidogenic enzymes (CYP11A1 and AKR1C3) was analyzed in CRPC patients with bone metastasis using a gene expression microarray dataset (GSE32269; Cai et al., 2013) 24.

### Plasmid construction

(a) Expression plasmids, pBABE-FLAG-ERRα, pcDNA3.1-FLAG-ERRα, pcDNA3.1-FLAG-ERRα truncated mutants (ΔDBD, ΔLBD-AF2 and ΔAF2), pcDNA3-PGC-1α/PGC-1α2×9 mutant, were constructed as described previously 21. Lentiviral plasmids pLKO.1-shERRα containing shRNA cassettes targeting ERRα were obtained from Dharmacon Inc. (b) Reporter gene plasmids. Luciferase reporter plasmids (CYP11A1 and AKR1C3) were constructed by cloning the respective gene promoter fragments, PCR-amplified from genomic DNA extracted from VCaP cells, into pGL3 basic vector. Sequences of the primers used for promoter cloning are listed in the Table S1. All plasmid constructs were confirmed by DNA sequencing before use.

### Viral transduction

For retrovirus production, pBABE-FLAG-ERRα or empty vector was transfected into PA317 packaging cells; for lentivirus production, pLKO.1- shERRα or pLKO-1-shScramble was transfected into 293FT cells as described previously 25, 26. LNCaP and DU145 cells were infected with pBABE-FLAF- ERRα or pLKO.1-shERRα, followed by antibiotic selection, for generation of stable ERRα-overexpressed or -knockdowned clones. All ERRα-transduced or shERRα-infected clones were validated by immunoblotting before use.

### *In vitro* cell growth analysis

Cells were seeded at density of 2 × 10^3^ cells/well in 96-well plates and cultured in normal medium with FBS or charcoal-stripped (CS) FBS medium for 1-7 days with fresh medium replaced every 3 days. Viable cells grown for every other days were determined by MTT assay as described previously 25. Briefly, cells were incubated with 100µl/well methylthiazolyldiphenyl-tetrazolium bromide (MTT, 0.5 mg/ml) in phenol red-free RPMI1640 medium for 4 h at 37ºC, followed by incubation with 100 µl/well SDS-HCl solution (10% SDS, 0.16% 6 M/10 mM HCl and 5% isobutanol) overnight in a CO_2_-free incubator at 37ºC to dissolve the formed formazan dyes. Absorbance *A*_570_ was measured in a microplate spectrophotometer. XCT790 and steroids were purchased from Sigma-Aldrich.

### CRPC xenograft tumors

CRPC xenograft model VCaP-CRPC was established based on the castration-resistant growth of tumors formed by subcutaneous inoculation of VCaP cells in castrated SCID mice as described previously 27. Tumor needle biopsies were acquired at same days when castration was performed and 8^th^-week post-castration (castration-relapse or CRPC) for mRNA and protein analysis.

### *In vivo* tumorigenicity assay

ERRα and vector-transduced clones (3 × 10^6^ cells suspended in 100 µl 1:1 growth medium-Matrigel mixture) were s.c. injected into the flank of intact male SCID mice and allowed to grow for 7-8 weeks for *in vivo* tumorigenicity evaluation as described previously 28. At about 9^th^ week, mice were orchiectomized for castration-resistance evaluation of clones following procedures as described previously 27. For *in vivo* C29 (Jiangsu Aikon Biopharmaceutical R&D Co. Ltd., China) treatment study, once the castration-relapsed VCaP/LNCaP-pBABE tumors regrew to the precastration sizes, tumor bearing mice were randomly assigned to daily intraperitoneal injection of vehicle (PBS containing 5.2% (v/v) of polyethylene glycol and 5.2% (v/v) of Tween 80) or C29 (10 mg/kg/day in vehicle) for 3 weeks 29. At the end of experiments, tumors were harvested and snap-frozen in liquid nitrogen for gene expression analyses, and androgen extraction and measurement.

### PCR and immunoblot analyses

#### Quantitative real-time qRT-PCR analysis

SYBR Green-based qRT-PCR was performed following procedures as described previously 25. Relative mRNA levels were determined by the comparative 2^-ΔΔCT^ method and normalized to β-actin. The specificity of primers was validated by the melting-curve detection. PCR cycle times or CT values ≥ 35 were considered to be very low levels or levels below detection. Information on primer sequences is listed in the Table S2.

#### Immunoblot analysis

An enhanced chemiluminescence method was used for immunosignals detection following procedures as described previously 30. Primary antibodies used are as follows: ERRα (ab76228, abcam), AKR1C3 (ab84327, abcam), CYP11A1 (D8F4F, Cell Signaling Technology) and β-actin (13E5, Cell Signaling Technology). Results were confirmed by at least three independent experiments.

### Molecular biology analyses

#### Chromatin immunoprecipitation (ChIP) -PCR assay

ChIP-PCR assay of CYP11A1 and AKR1C3 gene promoters was performed in VCaP cells following procedures as described previously 28 and using a commercial kit (SimpleChIP Enzymatic Chromatin IP kit, Cell Signaling Technology). Briefly, cross-linked and sonicated DNA samples obtained from VCaP cells were immunoprecipitated with anti-ERRα or control anti-IgG antibody followed by PCR analysis using primers specific for human *CYP11A1* and *AKR1C3* promoters listed in the Table S3.

#### Luciferase reporter assay

Dual luciferase reporter assay was performed in HEK293 cells co-transfected with reporter plasmids (pGL3-AKR1C3 or pGL3-CYP11A1), expression plasmids [pcDNA3-ERRα or its truncations, pcDNA3-PGC-1α (2×9)] and *Renilla* control reporter pRL-CMV using Lipofectamine 2000. Reporter activity was determined following procedures as described previously 25. All assays were repeated at least three times independently and results were presented as mean ± SD.

### Androgen measurement

Androgen levels in xenograft tumors were measured by ultra-high performance liquid chromatography coupled with electrospray ionization tandem mass spectrometry (UPLC-MS/MS) following procedures as described previously 27. Briefly, androgens were extracted from cultured cells and snap-frozen tumor samples homogenized in PBS. To evaluate the AKR1C3 activity for its conversion of 5α-androstanedione (5α-Adione) to dihydrotestosterone (DHT), cultured cells were first serum starved in phenol red-free medium with 1% charcoal-stripped serum for 48 h and then treated with androgen metabolite 5α-Adione (100 nmol/L) or 0.1 % ethanol (vehicle control) before androgen extraction. Cultured cells were collected, washed with PBS and lysed with cell lysis buffer. For frozen tumor samples, samples were weighed individually and homogenized in PBS. To serve as an internal controls, 50 pg of 3-deuteride-testosterone (T-3d) and 3-deuteride-dihydrotestosterone (DHT-3d) were added to the cell lysates or tumor homogenates. Each sample was extracted with 8 mL of diethyl ether, followed by freezing the aqueous phase in a dry-ice-ethanol bath. The residue was re-suspended in 0.5 mL of water and extracted with methylene chloride. Standards for T and DHT were prepared in parallel. The consequent oximes were analyzed by UPLC-MS/MS using an Agilent 1290 LC and Agilent 6460 Triple Quadrupole LC/MS system. Ions monitored were 289.4 > 109.2 and 292.4 > 109.2 for T and T-d3; and 291.2 > 255.4 and 294.2 > 258.4 for DHT and DHT-d3, respectively. The lower limits of quantification (LLOQ defined as a signal/noise ≥ 5) for T and DHT were 5 pg/ml and 10 pg/ml, respectively.

### Immunohistochemistry

Peroxidase immunohistochemistry (IHC) of ERRα and AKR1C3 was performed in a same construct of prostatic tissue microarray (TMA) slides containing benign prostatic hyperplasia (BPH, *n* = 3), and prostate cancer (*n* = 93) tissues using same antibodies as used in immunoblotting and following procedures as described previously 19. The ERRα and AKR1C3 immunosignals in stained sections were evaluated by a semiquantitative immunoreactivity scoring (IRS) method as described previously 31.

### Statistical analysis

All experiments were performed at least three times. Results are expressed as mean ± SD and analyzed by Student's *t*-test using the Graphpad 6.0 software with *P* values < 0.05 considered statistically significant.

## Results

### CRPC patients with high ERRα expression show poor clinical outcome

**Figure 1:**
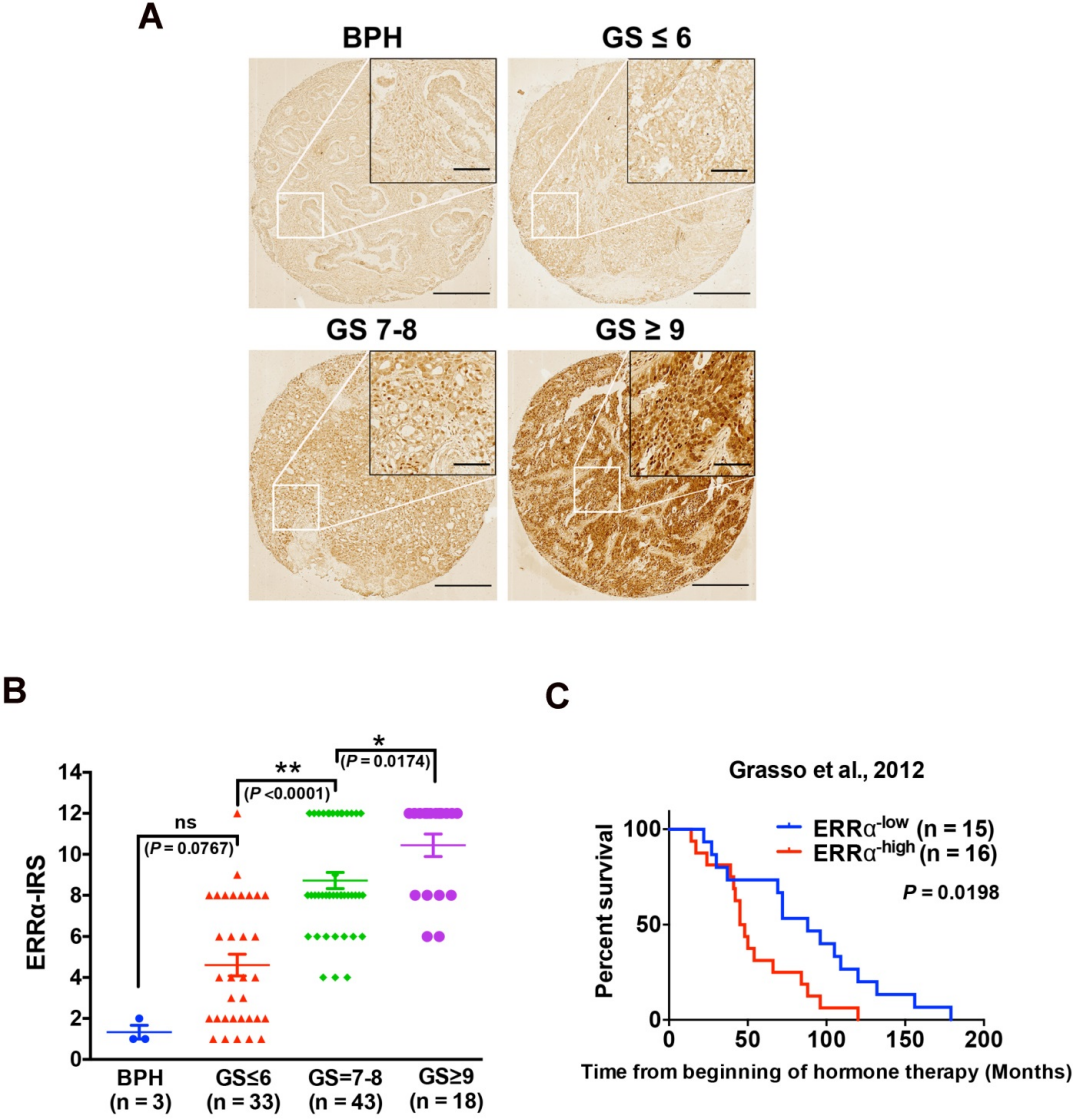
Up-regulation of ERRα is associated with CRPC. **(A)** ERRα immunohistochemistry. Representative micrographs of ERRα-immunostained tissue microarray spots of BPH and malignant lesions. A significant increase in malignant cells with positive and intense nuclear ERRα immunosignals was detected in adenocarcinoma lesions with higher Gleason scores. Magnification, ×40; scale bars, 250 μm. Insets show the enclosed areas at higher magnification. Magnification, ×200; scale bars, 50 μm. **(B)** ERRα immunoreactivity score analysis (ERRα‐IRS) performed on BPH and neoplastic prostatic tissues. Adenocarcinomas with higher Gleason scores (GS ≥ 7) showed significantly higher ERRα expression than BPH tissues. **(C)** Kaplan-Meier analysis of Grasso et al. 2012 study cohort (GSE35988) of CRPC revealed that higher ERRα mRNA expression was positively correlated with shortened overall survival of prostate cancer patients relapsed from hormone therapy.

Our previous studies show that ERRα performs oncogenic roles in the advanced growth of prostate cancer via its promotion of hypoxia growth adaptation 21 and also transcriptional regulation of TMPRSS2:ERG fusion gene 18. In order to elucidate further its clinical significance in the advanced progression of prostate cancer, we performed immunohistochemistry of ERRα in a prostatic tissue microarray slide containing benign prostatic hyperplasia (BPH, *n* = 3), and prostate cancer (*n* = 93) tissues of different Gleason scores (GS), and results showed that few cells showing positively-stained nuclei were detected in BPH and low-grade prostate cancer lesions, whereas significant increase of malignant cells with intense nuclear immunoreactivity was noticed in high-grade prostate cancer lesions. (Figure 1A). IRS analysis further confirmed that higher GS lesions (GS ≥ 7) exhibited significantly higher ERRα immunoreactivity scores as compared to lower GS lesions (GS ≤ 6) and BPH tissues (Figure 1B). In addition, we also analyzed the expression profile of ERRα in clinical samples of CRPC (including 31 CRPC patients totally) using a RNA-seq dataset from one CRPC cohort (Grasso et al., 2012) 23, which revealed that CRPC patients with higher expression of ERRα were positively correlated with shorter overall survival (Figure 1C). Together, these results showed increased expression of ERRα in advanced prostate cancers is associated with their poor clinical and pathological outcomes.

### Castration-relapse prostate cancer xenograft tumors expresses higher levels of ERRα and some key steroidogenic enzymes

To provide insights into whether ERRα would play a role in the castration-relapse growth in prostate cancer, we next examined the expression patterns of ERRα and also several major steroidogenic enzymes in an established castration-relapse xenograft tumor model VCaP-CRPC, in which AR-axis signaling was reactivated in castration-relapse VCaP xenograft tumors as compared to that in pre-castration 18, 27. Results of qRT-PCR and immunoblot analyses showed that the castration-relapse VCaP-CRPC xenograft tumors expressed higher levels of ERRα and also several key steroidogenic enzymes involved in androgen biosynthesis (Figures S1A-C). Fradet *et al.* previously analyzed a gene expression microarray dataset of CRPC with bone metastasis (GSE32269), showing that tumor tissues from metastatic CRPC displayed higher expression of ERRα than that in primary localized prostate cancer 22, 24. Analysis of the same dataset revealed that bone-metastatic CRPC tumor samples exhibited higher expression levels of two key steroidogenic enzymes, AKRIC3 and CYP11A1, than that in primary prostate cancer (Figures S1D-E). These results suggest that ERRα might play a role in intratumoral androgen biosynthesis in castration-relapse growth of prostate cancer.

### ERRα overexpression confers *in vitro* resistance to androgen-deprivation and antiandrogen in AR-positive prostate cancer cells

**Figure 2:**
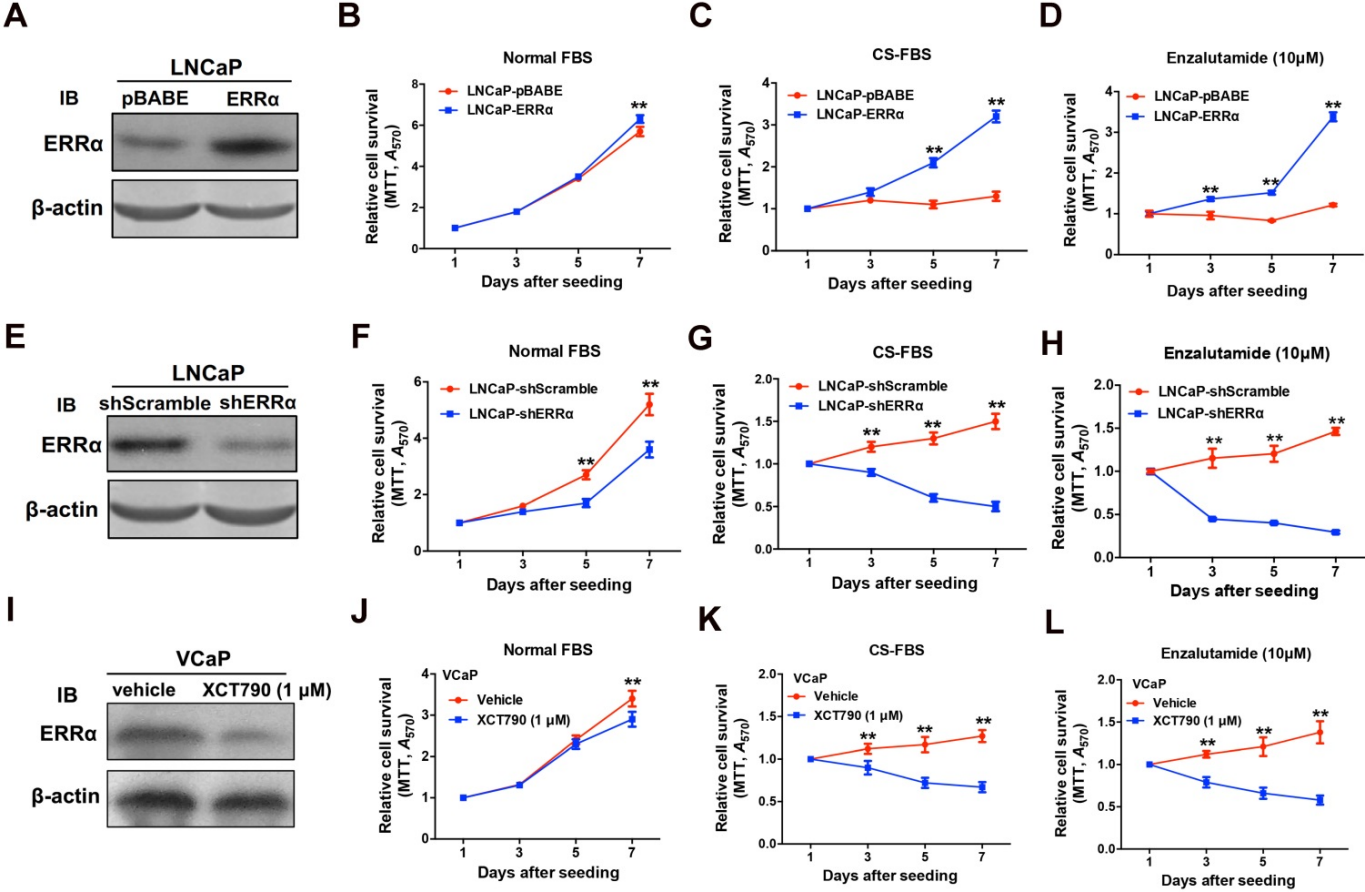
ERRα enhances *in vitro* growth resistance to androgen-deprivation and antiandrogen in prostate cancer cells. **(A-D)** ERRα-overexpression analysis. **(A)** Immunoblot validation of LNCaP-ERRα transduced clones. **(B-D)** *In vitro* growth responses of LNCaP-ERRα cells under androgen-deprivation culture condition (CS-FBS) and Enzalutamide treatment assayed by MTT. **(B)** Both LNCaP-pBABE and LNCaP-ERRα transduced cells grew at comparable rates in culture with normal FBS until Day-7. **(C and D)** However, when being cultured with CS-FBS or Enzalutamide, LNCaP-ERRα cells grew at normal rates, in sharp contrast to LNCaP-pBABE cells that did not grow. **(E-H)** ERRα-knockdown analysis. **(E)** Immunoblot validation of shERRα-transduced clones. **(F-H)** *In vitro* growth responses of LNCaP-shERRα cells toward cultures with CS-FBS and Enzalutamide as assayed by MTT. The LNCaP-shERRα cells grew at slower rate than the LNCaP-shScramble cells and did not grow in culture conditions with CS-FBS and Enzalutamide. **(I-J)** XCT790 treatment of VCaP cells. **(I)** Immunoblot analysis showed that XCT790 treatment could significantly reduce or abolish the protein level of ERRα in VCaP cells. **(J-L)** XCT790 treatment could significantly suppress the *in vitro* growth of VCaP cells cultured with CS-FBS or Enzalutamide. *, *P* < 0.05; **, *P* < 0.01 versus vector control LNCaP-pBABE, LNCaP-shScramble cells or vehicle treatment.

Since ERRα exhibited an up-regulation expression pattern in clinical CRPC tissues and CRPC xenograft tumors, we hypothesize that ERRα might play a supportive role in CRPC growth. To evaluate the functional impact of ERRα in prostate cancer growth, we generated both stable ERRα- overexpression and -knockdown transductants in two prostate cancer cell lines (including AR-positive: LNCaP; AR-negative: DU145) for *in vitro* and *in vivo* growth phenotype analyses (Figure 2 and Figure S2). *In vitro* growth analyses showed that when being culture in normal serum condition, the LNCaP-ERRα transduced clones proliferated at similar rate as the empty vector LNCaP-pBABE clones until Day-7. However, the LNCaP-ERRα cells exhibited significant/higher resistance to cultures with CS-FBS (mimicking androgen-deprivation condition) and antiandrogen (Enzalutamide) as compared to the LNCaP-pBABE cells that did not grow under these treatments (Figures 2A-D). Conversely, knockdown of ERRα in LNCaP-shERRα cells could substantially enhance the growth inhibition induced by androgen deprivation and antiandrogen as compared to LNCaP-shScramble cells (Figure 2E-H). Furthermore, treatment of another AR-positive VCaP cells with two ERRα inverse agonists XCT790 and Compound 29 (C29) could significantly sensitize the growth inhibition as exerted by CS-FBS and Enzalutamide (Figures 2I-L and Figures S3A-D) 32, 33. Interestingly, the suppressed growth of LNCaP and VCaP cells cultured with CS-FBS could be restored upon supplement with R1881 (a synthetic androgen agonist), and the change in proliferation rate (LNCaP-ERRα vs. LNCaP-pBABE and VCaP upon treatment with XCT790 vs. vehicle) in CS-FBS induced by enhancing or suppressing ERRα activity could be abolished by R1881 (Figures S3E-H). In addition, no significant change in growth proliferation was observed in AR-negative and ERRα-knockdown DU145-shERRα cells when they were cultured with CS-FBS or Enzalutamide until Day-7 (Figures S3I-L). Together, these results suggest that ERRα overexpression could enhance *in vitro* resistance to androgen deprivation and antiandrogen in AR-positive but not AR-negative prostate cancer cells.

### ERRα overexpression promotes *in vivo* castration-resistant tumorigenicity and enhances intratumoral androgen levels

**Figure 3:**
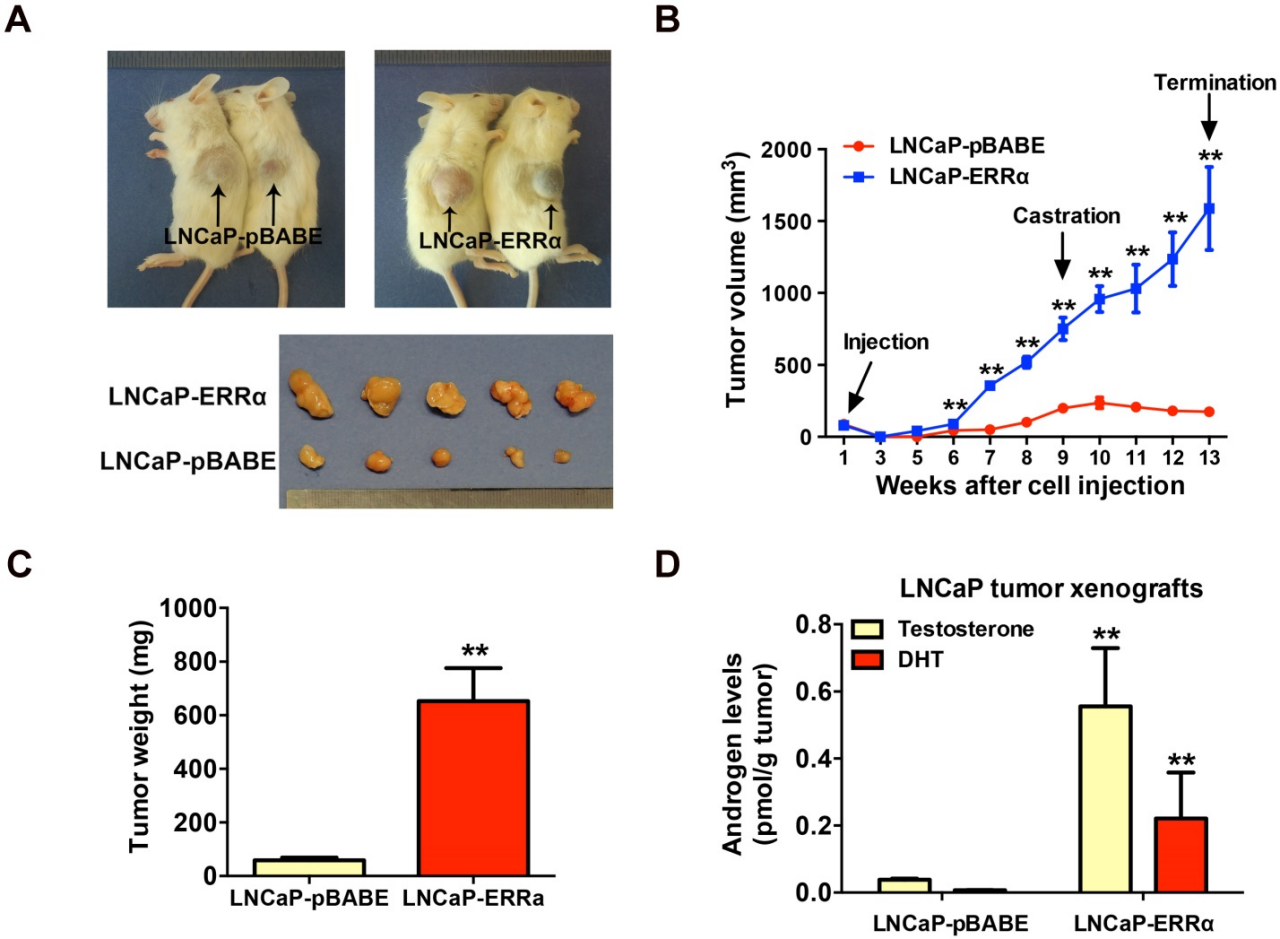
ERRα overexpression promotes *in vivo* castration-resistant growth capacity and enhances intratumoral androgen levels in LNCaP-ERRα-derived xenograft tumors. **(A)** Images show the representative castrated SCID mice bearing the xenograft tumors formed by LNCaP-ERRα and LNCaP-pBABE cells and the dissected xenograft tumors formed by the corresponding cells after 1-month growth in castrated host. **(B)** Growth curve shows the growth responses of LNCaP-ERRα and LNCaP-vector clones in intact host mice for initial 9 weeks followed by 4-week growth in same hosts after castration. LNCaP-ERRα clones formed larger tumors than vector clones in intact mice before castration and continued to grow aggressively in castrated hosts, as compared to tumors formed by LNCaP-pBABE clones that became atrophied after host castration. **(C)** Measurement of wet weights of xenograft tumors. LNCaP-ERRα clones formed tumors heavier than their vector counterpart in castrated hosts. **(D)** Measurement of androgens in xenograft tumors by LC-MS/MS. Significant higher levels of testosterone and DHT were detected in LNCaP-ERRα-derived tumors as compared to its vector counterpart. Data are represented as mean ± SD (n = 5) and analyzed by Students' *t*-test. *, *P* < 0.05; **, *P* < 0.01 versus vector control LNCaP-pBABE cells.

To further validate the enhanced resistance to androgen deprivation exerted by ERRα overexpression in prostate cancer cells, we then evaluated the *in vivo* tumorigenicity of LNCaP-ERRα transduced cells in castrated host mice. Results showed that the xenograft tumors formed by LNCaP-ERRα clones showed no response or resistance to castration and continued to grow aggressively, in sharp contrast to tumors formed by empty vector clones that stopped to grow and became shrunk in castrated hosts (Figures 3A-C). To further investigate the cause driving the *in vivo* castration-resistant growth capacity acquired in LNCaP-ERRα clones, we analyzed the intratumoral androgen levels (testosterone and DHT) in LNCaP- derived tumors grew in castrated hosts by UPLC-MS/ MS. Results showed that the LNCaP-ERRα-derived tumors grew in castrated hosts contained significant higher levels of androgens, as compared to their vector counterparts (Figure 3D). These results strongly suggest that the castration-resistant LNCaP- ERRα-derived tumors exhibited higher capacity of androgen production. Based on these results, we hypothesize that ERRα overexpression could increase *in situ* steroidogenesis or androgen biosynthesis in prostate cancer cells, leading to increased androgen levels in LNCaP-ERRα-derived tumors under castration condition, which promotes, at least in part, *in vivo* castration-resistant tumorigenicity.

### ERRα overexpression enhances expressions of key steroidogenic enzymes in prostate cancer cells

**Figure 4:**
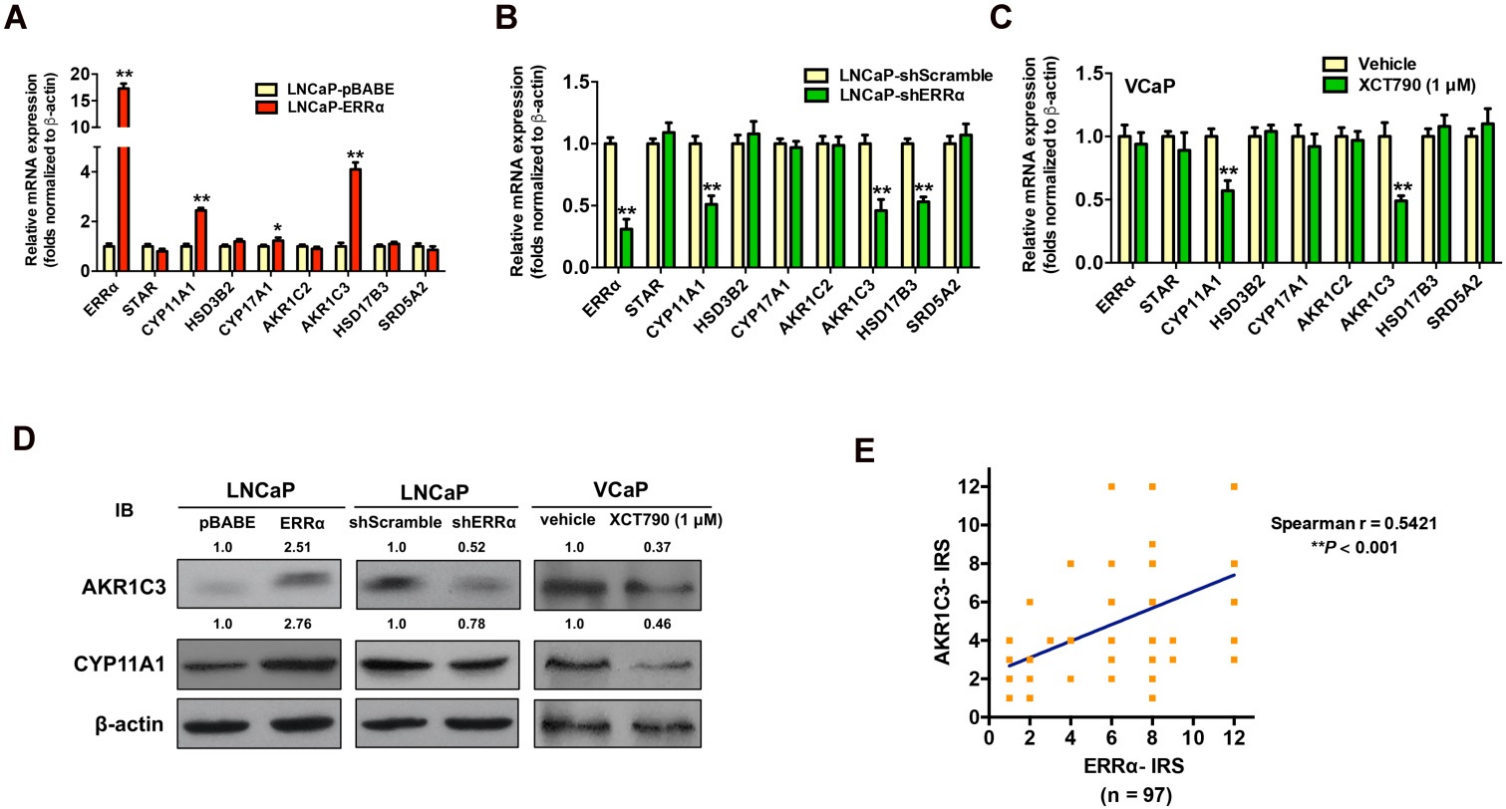
ERRα-overexpression enhances whereas its knockdown or suppression attenuates the expressions of some key steroidogenic enzymes in prostate cancer cells. **(A-C)** qRT-PCR analysis. **(A)** LNCaP-ERRα clones expressed higher transcript levels of CYP11A1 and AKR1C3 as compared to empty vector LNCaP-pBABE clones. **(B)** ERRα-knockdown in LNCaP-shERRα cells. ERRα-knockdown could significantly suppress the mRNA levels of CYP11A1 and AKR1C3 in LNCaP-shERRα clones as compared to their shScramble clones. **(C)** XCT790 treatment. XCT790 treatment could significantly suppress the mRNA levels of CYP11A1 and AKT1C3 in VCaP cells. *, *P* < 0.05; **, *P* < 0.01 versus LNCaP-pBABE cells, LNCaP-shScramble cells or vehicle treatment. **(D)** Immunoblot analysis of AKR1C3 and CYP11A1 in LNCaP-ERRα cells, LNCaP-shERRα cells and XCT790-treated VCaP cells. ERRα-overexpression enhanced AKR1C3 and CYP11A1 expression in LNCaP-ERRα cells whereas ERRα-knockdown and XCT790 treatment suppressed AKR1C3 and CYP11A1 expression in LNCaP and VCaP cells, respectively. **(E)** Linear regression analysis between ERRα and AKR1C3 immunoreactivity scores (IRS). Results showed ERRα and AKR1C3 manifested a positive correlation in their IRS.

To verify our hypothesis, we next examined the expression profiles of key steroidogenic enzymes involved in androgen biosynthesis in AR-positive prostate cancer cells with either stable ERRα- overexpression or -knockdown. qRT-PCR analysis of ERRα- or shERRα-transduced prostate cancer clones showed that the LNCaP-ERRα transduced clones expressed significant higher mRNA levels of three steroidogenic enzymes (CYP11A1, AKR1C3 and CYP17A1), whereas the LNCaP-shERRα transduced clones exhibited significant reduced levels of three steroidogenic enzymes (CYP11A1, AKR1C3 and HSD17B3) (Figures 4A and B). qRT-PCR analysis showed that suppression of ERRα activity by XCT790 or C29 could significantly reduce the transcript levels of CYP11A1 and AKR1C3 in another AR-positive VCaP cells (Figure 4C and Figure S4A). Immunoblot analysis also validated that overexpression of ERRα could enhance AKR1C3 and CYP11A1 expression in LNCaP-ERRα cells whereas ERRα-knockdown and XCT790 treatment could reduce their expression in LNCaP and VCaP cells, respectively (Figure 4D). The regulation of AKR1C3 and CYP11A1 expression by ERRα was also observed in AR-positive LNCaP and VCaP cells under androgen-deprivation culture condition (CS-FBS) and in AR-negative DU145 cells (Figures S4B-D). Furthermore, we performed AKR1C3 IHC on the same construct of prostatic tissue TMA slides used for ERRα IHC (Figure S4E). IRS analysis revealed that higher GS lesions (GS ≥ 7) exhibited significantly higher AKR1C3 IRS scores as compared to lower GS lesions (GS ≤ 6) and BPH tissues (Figure S4F), suggesting that ERRα and AKR1C3 manifested a positive correlation in their immunoreactivity scores (Figure 4E). Finally, the expression of scavenger receptor B1 (SR-B1) encoded by *SCARB1* gene, which is reported to facilitate the precursor cholesterol uptake as needed to drive steroidogenic and non-steroidogenic biogenic pathways in prostate cancer 34, showed no change in either LNCaP-ERRα or LNCaP-shERRα cells, suggesting that SR-B1-mediated precursor cholesterol uptake might not be affected by ERRα (Figure S4G). These results showed that ERRα overexpression exhibited a positive correlation with the up-regulation of two steroidogenic rate-limiting enzyme genes, CYP11A1 and AKR1C3, involved in androgen biosynthesis in prostate cancer cells, suggesting that ERRα could function to promote the castration-resistant growth via the up-regulation of steroidogenic enzymes involved in androgen biosynthesis.

### ERRα can directly transactivate steroidogenic enzyme genes AKR1C3 and CYP11A1 in prostate cancer cells

**Figure 5:**
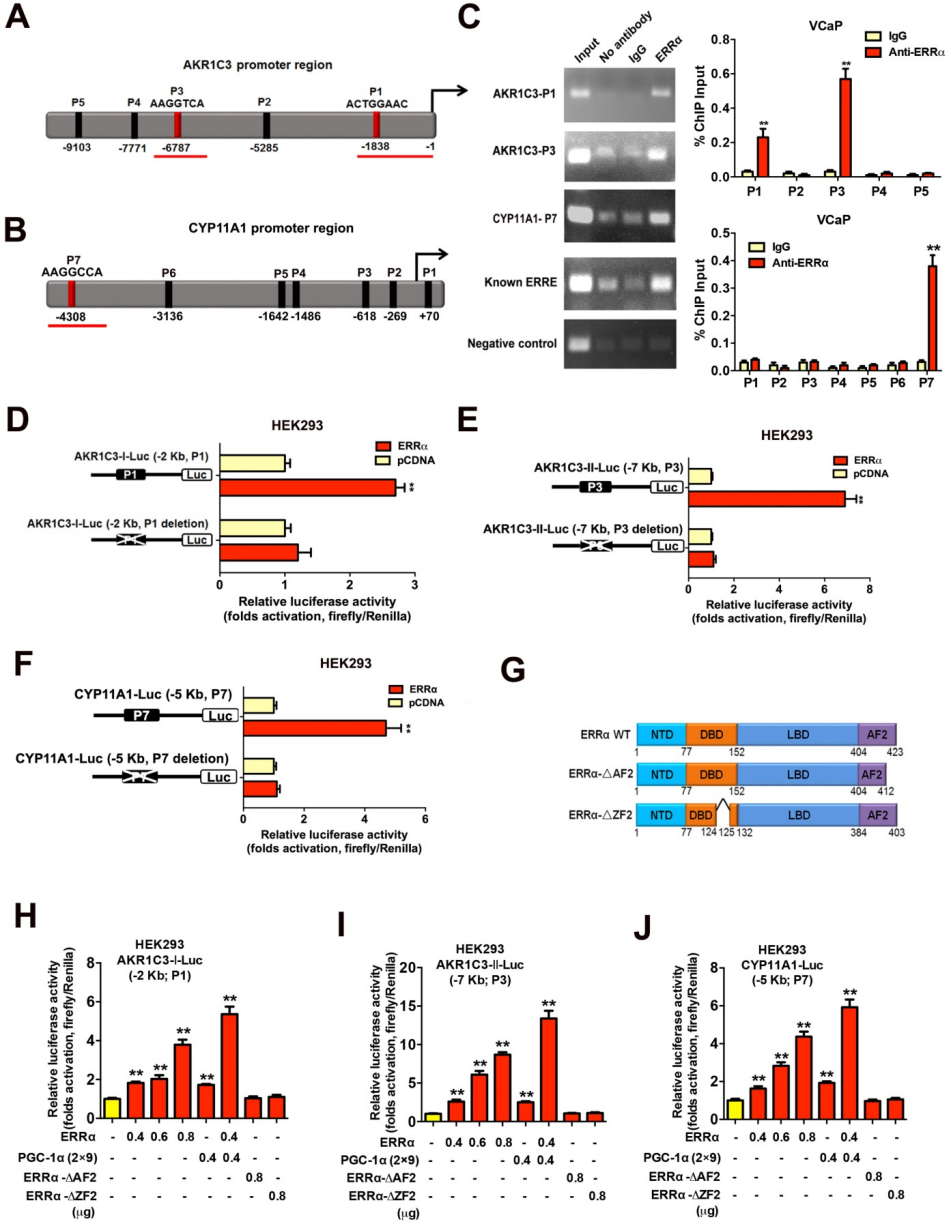
Direct transactivation of AKR1C3 and CYP11A1 genes by ERRα in prostate cancer cells. **(A and B)** Schematic diagrams depict the putative ERRα-binding sites (ERREs), as predicted by the online program MatInspector (https://www.genomatrix.de), located in the **(A)** AKR1C3 (P1-P5) and **(B)** CYP11A1 (P1-P7) gene promoter/regulatory regions. **(C)** ChIP-PCR assay of AKR1C3 and CYP11A1 gene promoters performed in VCaP prostate cancer cells. Results validated that two ERRE sites (P1 and P3) located respectively at 1.8 kb and 6.8 kb upstream of the transcription start site of AKR1C3 promoter (upper graph), and one ERRE site (P7) located at 4.3 kb upstream of the CYP11A1 promoter (lower graph), were enriched of ERRα. **, *P* < 0.01 versus non-immune IgG-treated DNA. **(D-F)** Luciferase reporter assay of AKR1C3-Luc and CYP11A1-Luc reporters performed in ERRα-transfected HEK293 cells. The AKR1C3-Luc and CYP11A1-Luc reporter constructs, containing inserts of ERRα promoter/regulatory regions, could be significantly transactivated by the transfected ERRα. Deletion of identified ERREs in the reporters (P1 and P3 in AKR1C3-Luc; P7 in CYP11A1-Luc) abolished or significantly reduced the ERRα-induced transactivation. **(G)** Schematic diagram shows the functional domains of wild-type ERRα protein and two of its truncated mutants generated. **(H-J)** Luciferase reporter assays of AKR1C3-Luc and CYP11A1-Luc reporters performed in HEK293 cells. The AKR1C3-Luc and CYP11A1-Luc reporters could be dose-dependently transactivated by ERRα and further potentiated by co-transfection with co-regulator PGC-1α (2×9), but not by ERRα-ΔAF2 and ERRα-ΔZF2 truncated mutants. *, *P* < 0.05; **, *P* < 0.01 versus empty vector pcDNA3.1. Data are presented as mean ± SD (n = 3) and analyzed by Students' *t*-test.

We next sought to determine whether the up-regulation of the two androgen biosynthetic enzyme genes AKR1C3 and CYP11A1 could be the result of direct targeting of ERRα in prostate cancer cells. Promoter sequence analysis predicted multiple potential ERRα-binding sites (ERREs) present in the promoter or regulatory regions of AKR1C3 (17β-hydroxysteroid dehydrogenase 5) and CYP11A1 (cholesterol desmolase) genes respectively (Figures 5A and B). ChIP-PCR assay validated that two DNA fragments of AKR1C3 gene (designated as P1 and P3 sites located at about -1,838 bp and -6,787 bp upstream) and one DNA fragment of CYP11A1 gene (designated as P7 site located at -4,308 bp upstream) could be PCR-amplified in the ERRα-immunoprecipitated DNA extracted from the VCaP cells (Figure 5C). Reporter gene assays performed in HEK293 cells confirmed that the AKR1C3 and CYP11A1 gene promoters-driven reporter constructs could be dose-dependently transactivated by the transfected intact ERRα, with further potentiation by co-transfection with an ERRα-specific co-regulator PGC-1α (2×9) (Figures 5D-J). However, deletion of the identified ERREs in the AKR1C3 or CYP11A1 promoter inserts in reporters abolished their transactivation by ERRα, further suggesting that these ERREs were essential for the ERRα-mediated transactivation. Further truncation analysis of the functional domains of ERRα demonstrated that the AKR1C3-Luc and CYP11A1-Luc reporters could only be transactivated by the intact ERRα but not its DBD/AF2-deleted mutants. Together, these results suggest that ERRα could directly transactivate the AKR1C3 and CYP11A1 genes through its direct binding to multiple binding motifs in their promoter or regulatory regions.

### ERRα-mediated AKR1C3 expression enhances the DHT production in prostate cancer cells

**Figure 6:**
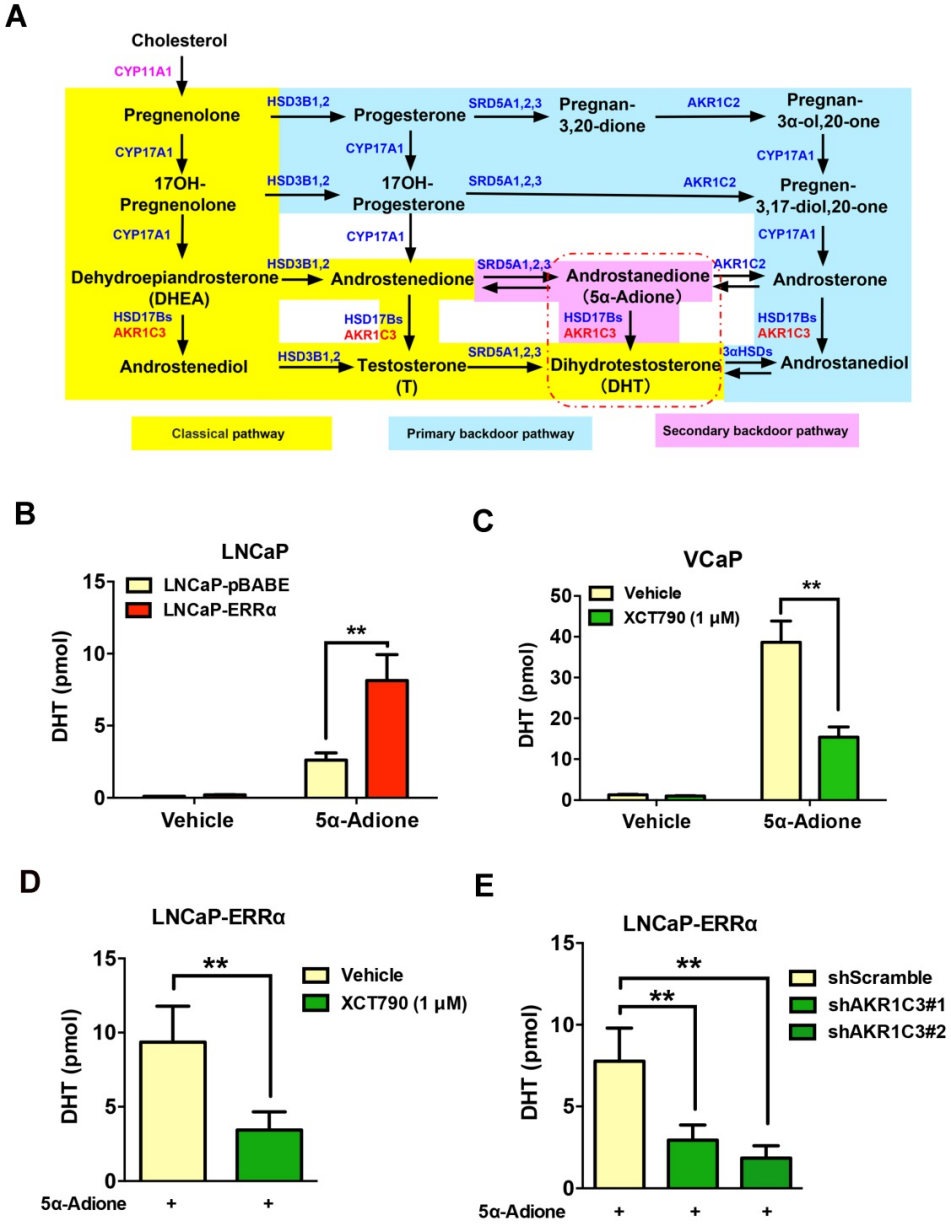
ERRα-overexpression can promote while its suppression can attenuate DHT production in prostate cancer cells. **(A)** Diagram shows the classical (canonical or front-door) and alternative (back-door) pathways of androgen biosynthesis. Androgens are synthesized from cholesterol via multiple enzymatic steps. CYP11A1 is responsible for converting cholesterol to pregnenolone by side chain cleavage of cholesterol. Pregnenolone is then converted to dehydroepiandrosterone (DHEA) and androstenedione by CYP17A1. The classical pathway for testosterone biosynthesis involves conversion of major adrenal DHEA and androstenediol to testosterone in the testis, followed by 5α-reduction of testosterone to dihydrotestosterone (DHT) by 5α-reductases (SRD5As). On the other hand, DHT biosynthesis via 5α-reduction of upstream steroids bypassing testosterone can be achieved by two other back-door pathways. In the primary backdoor pathway, 17OH-progesterone is 5α- and 3α-reduced by SRD5As and AKR1C2 before the 17,20-lyase reaction of CYP17A1, yielding the androsterone and then to androstanediol by HSD17Bs and AKR1C3. In the secondary backdoor pathway, androstenedione is converted to 5α-androstenedione (5α-Adione) by SRD5As and then to DHT by HSD17Bs and AKR1C3. Through these backdoor pathways, DHT is synthesized without using testosterone as intermediate. **(B and C)** UPLC-MS/MS measurement of DHT in LNCaP-ERRα cells and XCT790-treated VCaP cells. Results showed that upon supplement with 5α-Adione, LNCaP-ERRα cells contained significant higher level of DHT than the empty vector LNCaP-pBABE cells. On the other hand, suppression of ERRα activity by XCT790 could significantly reduce the DHT level in 5α-Adione-supplemented VCaP cells. **(D and E)** UPLC-MS/MS measurement of DHT in LNCaP-ERRα cells upon XCT790 treatment and shRNA-mediated knockdown of AKR1C3. Results showed that ERRα overexpression-induced increase of DHT biosynthesis could be abolished by either XCT790 treatment or AKR1C3 knockdown. *, *P* < 0.05; **, *P* < 0.01 versus empty vector pBABE or vehicle.

It is known that AKR1C3 is a key steroidogenic enzyme involved in the biosynthesis of DHT via its reduction of androgen intermediates to DHT bypassing testosterone biosynthesis, in an alternative androgen biosynthesis pathway known as the “backdoor” pathway (Figure 6A) 10, 35. Powell *et al.* revealed that testosterone precursors androstenedione and androstenediol were converted to DHT at very low levels in VCaP cells, suggesting that the 5α-Adione pathway (secondary backdoor pathway) has a more profound impact on DHT biosynthesis in prostate cancer cells 36. Since the expression of CYP11A1 is rather low in prostate cancer cells and hardly detectable in clinical prostate cancer tissues, we next manly investigated the significance of ERRα-enhanced AKR1C3 expression or activity in the DHT biosynthesis in AR-positive prostate cancer cells. UPLC-MS/MS analysis showed that the LNCaP-ERRα transduced cells displayed higher capacity of DHT production upon supplement with precursor 5α-androstanedione (5α-Adione), whereas such 5α-Adione-enhanced DHT production capacity was significantly attenuated by XCT790 treatment in VCaP and LNCaP cells (Figures 6B-C and Figures S5A-D). Importantly, the increased DHT biosynthesis induced by ERRα overexpression could be abolished by either XCT790 treatment or shRNA-mediated knockdown of AKR1C3 (Figures 6D-E and Figures S5E-F). Furthermore, XCT790 treatment and AKR1C3 silencing could re-sensitize LNCaP-ERRα cells to enzalutamide or culture condition with CS-FBS (Figures S5G-H), suggesting that AKR1C3, at least partially, was involved in the ERRα overexpression- mediated *in vitro* resistance to antiandrogen and androgen-deprivation condition. Overall, these results suggest that ERRα could function to promote the intratumoral DHT biosynthesis in prostate cancer or CRPC via its direct regulation of AKR1C3 expression and also pharmacological suppression of ERRα activity could reduce the DHT production in prostate cancer cells.

### ERRα can function to activate AR signaling in prostate cancer cells

**Figure 7:**
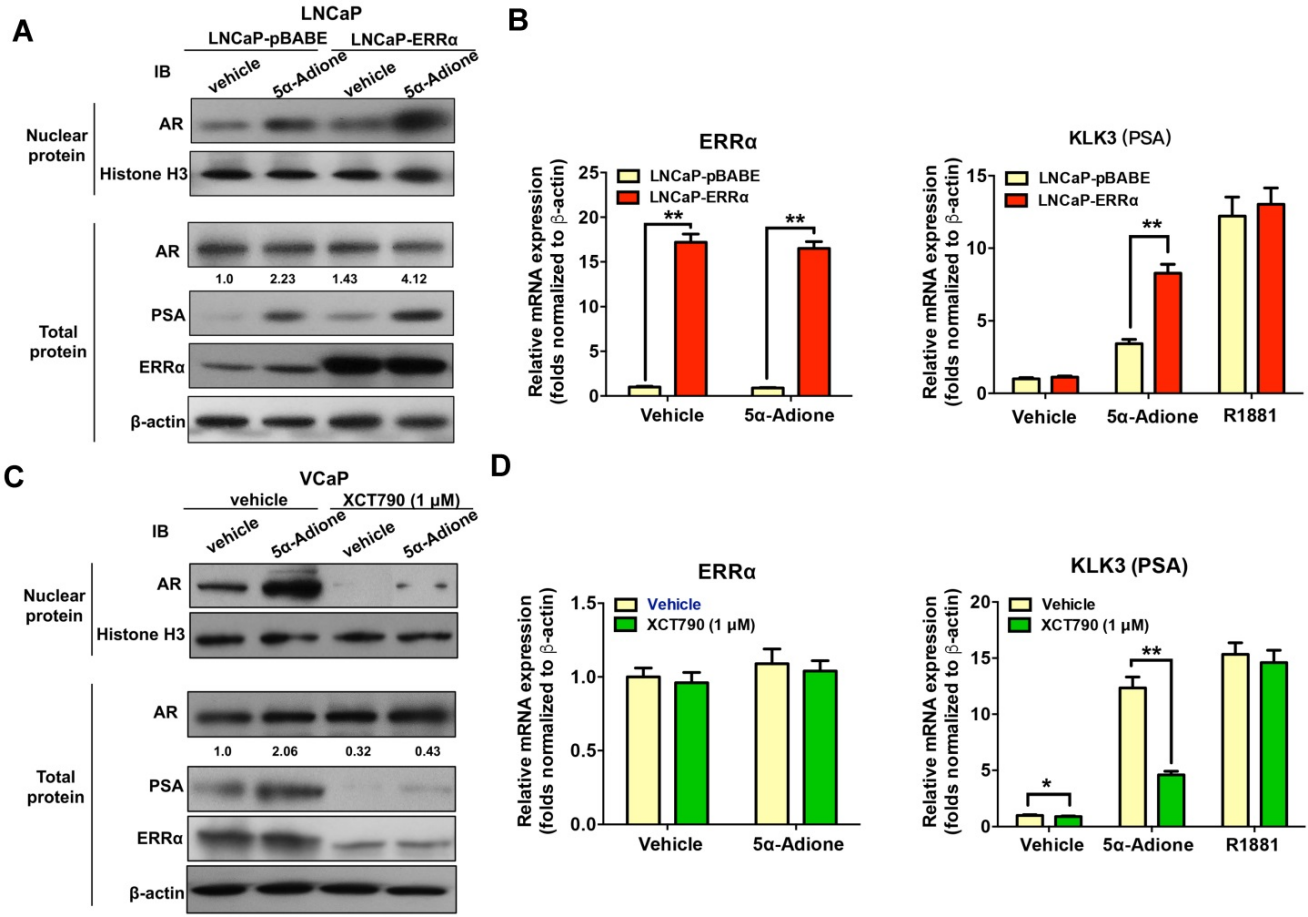
ERRα functions to activate AR signaling in prostate cancer cells. Cultured cells were first serum-starved in phenol red-free medium with 1% CS-FBS for 48 hours and then treated with androgen metabolite 5α-Adione (100 nmol/L) or vehicle control (0.1% ethanol) for 24 hours before mRNA or protein analyses. **(A)** Immunoblot analysis of AR and PSA in LNCaP-pBABE and LNCaP-ERRα transduced cells. Results showed that supplement with 5α-Adione could enhance the nuclear AR (with no change in total AR level) and PSA expression levels in both LNCaP-pBABE and LNCaP-ERRα cells, with significant higher levels in LNCaP-ERRα cells. **(B)** qRT-PCR analysis of ERRα and KLK3 (PSA) expression in 5α-Adione-supplemented LNCaP-pBABE and LNCaP-ERRα transduced cells. Supplement with 5α-Adione induced no change in mRNA levels of ERRα in both LNCaP-pBABE and LNCaP-ERRα cells. However, 5α-Adione supplement could significantly increase PSA mRNA levels in both LNCaP-pBABE and LNCaP-ERRα cells, with significant higher levels in LNCaP-ERRα cells. Supplement with R1881 induced increase of PSA mRNA level**s** in both LNCaP-pBABE and LNCaP-ERRα cells at same levels.** (C)** Immunoblot analysis of AR and PSA in VCaP cells treated with 5α-Adione and XCT790. Results showed that suppression of ERRα activity by XCT790 could significantly reduce or abolish the protein levels of nuclear AR, PSA and ERRα in VCaP cells with or without 5α-Adione supplement. **(D)** qRT-PCR analysis of ERRα and PSA expression in VCaP cells treated with 5α-Adione and XCT790. Results showed that XCT790 treatment did not affect the mRNA levels of ERRα, but significantly suppressed the mRNA levels of KLK3 in VCaP cells supplemented with 5α-Adione. Supplement with R1881 induced increase of PSA mRNA level**s** in VCaP cells upon treatment with XCT790 or vehicle at same levels. **, *P* < 0.01 versus empty vector pBABE transduced cells or vehicle.

Finally, we investigated the functional significance of ERRα-mediated up-regulation of AKR1C3 in activation of AR signaling in prostate cancer cells. Immunoblot analysis of AR and its target PSA in prostate cancer cells showed that supplement with an androgen precursor 5α-Adione could enhance the levels of nuclear AR level (without change in total AR level) and PSA in both LNCaP-pBABE and LNCaP-ERRα transduced cells, with significant higher levels in LNCaP-ERRα cells (Figure 7A). qRT-PCR analysis revealed that 5α-Adione supplement could significantly increase the PSA mRNA levels in both LNCaP-ERRα and LNCaP-pBABE transduced cells, with much higher levels induced in LNCaP-ERRα cells (Figure 7B). We also confirmed that suppression of ERRα activity by XCT790 could significantly reduce or abolish the levels of AR (nuclear) and PSA in AR-positive VCaP cells supplemented with or without 5α-Adione (Figures 7C and D), accompanied with the decreased ERRα protein expression upon XCT790 treatment. Similar reductions of levels of AR (nuclear) and PSA were also confirmed in stable ERRα-knockdowned LNCaP-shERRα cells with or without 5α-Adione (Figure S6). Together, these results demonstrate the significance of ERRα-mediated up-regulation of AKR1C3 in the activation of AR signaling in prostate cancer cells through the enzymatic role of AKR1C3 in the backdoor pathway of androgen biosynthesis. Overall, these results suggest that ERRα could play a role in activation of AR signaling in prostate cancer cells via its regulation of AKR1C3 expression.

### Pharmacological targeting of ERRα can suppress castration-resistant growth of prostate cancer *in vivo*

**Figure 8:**
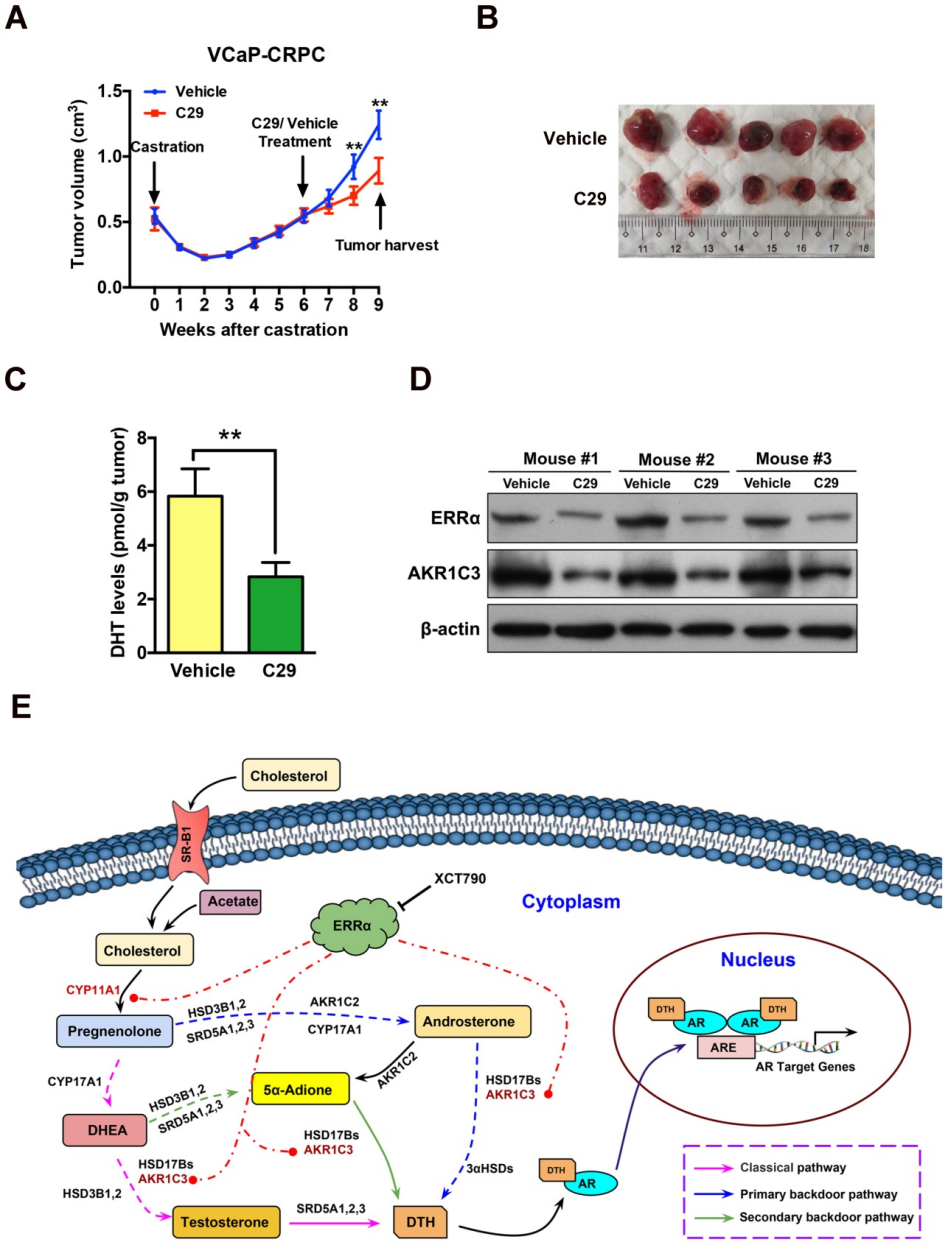
ERRα inverse agonist C29 suppresses castration-resistant growth of prostate cancer *in vivo*. **(A)** Growth curve of VCaP-CRPC xenograft tumors upon C29 or vehicle treatment. Once the castration-relapsed VCaP-CRPC tumors regrew to sizes as that in pre-castration at 6-7 week post-castration, tumor-bearing mice were randomly assigned to intraperitoneal injections of C29 or vehicle for additional 3 weeks. Results showed that C29 could significantly retard the castration-relapsed growth of VCaP-CRPC tumors as compared to vehicle. **(B)** Images show the representative dissected VCaP-CRPC xenograft tumors upon 3-week treatment with C29 or vehicle in castrated host. **(C)** Measurement of DHT in VCaP-CRPC xenograft tumors by LC-MS/MS. Significant reduction of DHT levels was detected in tumors upon C29 treatment as compared vehicle.** (D)** Immunoblot analysis. VCaP-CRPC tumors upon C29 treatment expressed lower protein levels of ERRα and AKR1C3 as compared to vehicle. **, *P* < 0.01 versus vehicle. **(E)** Schematic diagram depicts the hypothetical role of ERRα in the promotion of intratumoral androgen biosynthesis and reactivation of AR signaling in CRPC via its direct transactivation of some key steroidogenic enzyme genes.

To evaluate the therapeutic significance and application value of targeting ERRα in CRPC, we assessed the anti-tumor effect of suppression of ERRα in the VCaP-CRPC model by intraperitoneal injection of C29 or vehicle. The results showed that C29 could significantly prevent the castration-relapsed tumor growth as compared to vehicle, accompanied by decrease of intratumoral DHT production and expression of ERRα and AKR1C3 (Figures 8A-D). A similar inhibitory effect as exerted by C29 treatment was also observed in castration-resistant LNCaP- pBABE and LNCaP-ERRα xenografts, in which the C29-induced growth inhibition was more significant in LNCaP-ERRα xenografts as compared to that in LNCaP-pBABE xenografts (Figures S7A-D). These results suggest that ERRα is a potential therapeutic target of CRPC and pharmacological suppression of ERRα could potentiate the sensitivity of prostate cancer cells to hormone or AR-axis targeting therapy.

## Discussion

Enhanced intratumoral androgen biosynthesis caused by up-regulation of androgen synthesis enzymes in CRPC tissues is regarded as one of the key factors responsible for the reactivation of AR signaling in CRPC and also its relapse growth. Based on this, selective inhibition of the key enzyme in early steroidogenesis, CYP17A1 by abiraterone acetate, is developed as a novel androgen-deprivation therapy and its clinical use can prolong the survival of patients with locally advanced or metastatic CRPC 37-39. However, resistance to abiraterone acetate still inevitably occurs in CRPC patients 40. A few studies on CRPC xenograft models show that resistance to abiraterone acetate may involve multiple mechanisms, including up-regulation of CYP17A resulting in enhanced *de novo* androgen biosynthesis 9, 41, induction of ligand-independent AR splice variants and progesterone-sensitive mutated AR 9, 41, and acquisition of mutated steroidogenic enzyme 3βHSD1 that can accelerate the conversion of adrenal steroid DHEA to DHT 42. On the other hand, the mechanisms involved in the up-regulation of androgen synthesis enzymes in CRPC and also resistance to abiraterone still remain largely unknown. Here we show that the orphan nuclear receptor ERRα could be a key factor for the transcriptional regulation of some key androgen synthesis enzymes in prostate cancer cells and its overexpression would be responsible for the enhanced intratumoral androgen biosynthesis in CRPC.

In this study, we showed that ERRα displayed a higher expression pattern in advanced prostate cancer and a CRPC xenograft model VCaP-CRPC, and its up-regulation was positively correlated with poor clinical outcome. These results validate the previous reports by us and others that ERRα exhibits an up-regulation pattern in advanced prostate cancer and also its usefulness as a poor prognostic marker for prostate cancer 18-20, 22. Furthermore, our present study showed that its overexpression could confer *in vitro* resistance to androgen-deprivation and antiandrogen, and also *in vivo* castration-resistant growth capacity of AR-positive cancer cells. Our results suggest that overexpression of ERRα could play a significant role in the development and progression of CRPC. However, the mechanisms responsible for the up-regulation of ERRα in prostate cancer are still unclear. Genomic amplification of *ESRRA* gene and down-regulation of an ERRα-targeting microRNA miR-125a are shown to be responsible for the up-regulation of ERRα in oral squamous cell carcinoma 43, 44. Indeed, genotype with amplification at chromosome 11q13.1, where the *ESRRA* gene is located, is detected in localized prostate cancer tumors by comparative genomic hybridization (CGH) analysis and such genotype is shown as a positive predictor of post-operative disease recurrence and metastasis 45. Recently, we demonstrate that the transcription factor ERG, expressed by the TMPRSS2:ERG fusion gene, can directly transactivate the *ESRRA* gene in advanced prostate cancer 18. These studies provide indirect evidence that genetic factors, such as gene amplification and transactivation by transcription factors, could be one of the mechanisms responsible for the increased expression of ERRα in advanced prostate cancer.

One key finding we showed in this study is that overexpression of ERRα could confer *in vitro* resistance to androgen-deprivation and *in vivo* castration-resistant growth capacity in AR-positive prostate cancer cells via a mechanism of direct transactivation of some key androgen synthetic enzyme genes, leading to enhanced intracellular *de novo* androgen production in prostate cancer cells. In a recent study, we also demonstrate that another nuclear receptor LRH-1 (*NR5A2*), which exhibits an increased expression in CRPC, can play a supportive role in intratumoral androgen biosynthesis in CRPC via a similar mechanism of direct transactivation of key androgen synthetic enzyme genes 27. Interestingly, comparing their specific targets, it is noted that ERRα can target the CYP11A1 and AKR1C3 (involved in the *de novo* and backdoor pathways of DHT biosynthesis), whereas LRH-1 can target the STAR, CYP11A, CYP17A1 and HSD3B2 (involved in similar pathways except the secondary backdoor pathway), suggesting that the two nuclear receptors may perform differential roles in biosynthesis of DHT via their regulation of different steroidogenic enzymes involved in the intratumoral androgen biosynthesis in CRPC. These results also implicate that the prostate cancer cells in CRPC can shift to employ alternative pathways of DHT biosynthesis via their flexible or adaptive transcriptional control of androgen synthestic enzyme genes by different nuclear receptors including ERRα and LRH-1.

Although ERRα is regarded as an orphan nuclear receptor, its transactivation activity can be targeted or modulated by synthetic ligands, such as the inverse agonist XCT790. One noteworthy finding of this study is that ERRα could be druggable as the intracellular DHT production and AR signaling in prostate cancer cells could be significantly attenuated or suppressed by treatment with XCT790, likely via its direct repression on AKR1C3 enzyme and reduction of ERRα protein level. This result also implicates that besides inhibition of activities of steroidogenic enzymes (e.g. CYP17A1 targeting abiraterone), targeting their upstream regulators (such as ERRα) could be a potential therapeutic strategy for the suppression of intratumoral androgen biosynthesis in CRPC.

Recently, it is shown that the oncogenic transcription factor ERG, expressed by the TMPRSS2:ERG fusion gene, could upregulate the AKR1C3 expression in prostate cancer 36. Our recent study also shows that ERRα and ERG can synergistically regulate each other at the transcriptional level and both form a reciprocal regulatory loop to promote the advanced growth of prostate cancer. Together, these findings suggest that besides CYP17A1, AKR1C3 and ERRα could also be the potential therapeutic targets in the management of CRPC.

## Conclusions

In summary, our present study shows that the nuclear receptor ERRα could perform a supportive role in the intratumoral androgen biosynthesis in prostate cancer via its direct transactivation of at least two key steroidogenic enzyme genes CYP11A1 and AKR1C3, and through this regulation it could help to promote the advanced growth of CRPC by activation of AR signaling (Figure 8E). Our study also provides a novel insight that targeting ERRα could be a potential androgen-deprivation strategy for the management of CRPC as pharmacological suppression of its activity could help to attenuate the intracellular production of DHT and AR signaling in prostate cancer cells.

## Supplementary Material

Supplementary figures and tables.

## Abbreviations

5α-Adione: 5α-androstanedione
AR: androgen receptor
CGH: comparative genomic hybridization
ChIP: chromatin immunoprecipitation
CRPC: castration-resistant prostate cancer
CS: charcoal- stripped
DHEA: dehydroepiandrosterone
DHT: dihydrotestosterone
ERRα: estrogen-related receptor alpha
GEO: gene expression omnibus
qRT-PCR: quantitative real-time PCR
RNA Sequencing: RNA-Seq
T: testosterone

## References

[1] Scher HI, Solo K, Valant J, Todd MB, Mehra M (2015). Prevalence of Prostate Cancer Clinical States and Mortality in the United States: Estimates Using a Dynamic Progression Model. PLoS One.

[2] Nadiminty N, Gao AC (2012). Mechanisms of persistent activation of the androgen receptor in CRPC: recent advances and future perspectives. World J Urol.

[3] Schweizer MT, Yu EY (2015). Persistent androgen receptor addiction in castration-resistant prostate cancer. J Hematol Oncol.

[4] Egan A, Dong Y, Zhang H, Qi Y, Balk SP, Sartor O (2014). Castration-resistant prostate cancer: adaptive responses in the androgen axis. Cancer Treat Rev.

[5] Mitsiades N (2013). A road map to comprehensive androgen receptor axis targeting for castration-resistant prostate cancer. Cancer Res.

[6] Graham L, Schweizer MT (2016). Targeting persistent androgen receptor signaling in castration-resistant prostate cancer. Med Oncol.

[7] Locke JA, Guns ES, Lubik AA, Adomat HH, Hendy SC, Wood CA (2008). Androgen levels increase by intratumoral de novo steroidogenesis during progression of castration-resistant prostate cancer. Cancer Res.

[8] Stanbrough M, Bubley GJ, Ross K, Golub TR, Rubin MA, Penning TM (2006). Increased expression of genes converting adrenal androgens to testosterone in androgen-independent prostate cancer. Cancer Res.

[9] Cai C, Chen S, Ng P, Bubley GJ, Nelson PS, Mostaghel EA (2011). Intratumoral de novo steroid synthesis activates androgen receptor in castration-resistant prostate cancer and is upregulated by treatment with CYP17A1 inhibitors. Cancer Res.

[10] Chang KH, Li R, Papari-Zareei M, Watumull L, Zhao YD, Auchus RJ (2011). Dihydrotestosterone synthesis bypasses testosterone to drive castration-resistant prostate cancer. Proc Natl Acad Sci U S A.

[11] Ferraldeschi R, Welti J, Luo J, Attard G, de Bono JS (2015). Targeting the androgen receptor pathway in castration-resistant prostate cancer: progresses and prospects. Oncogene.

[12] Montgomery RB, Mostaghel EA, Vessella R, Hess DL, Kalhorn TF, Higano CS (2008). Maintenance of intratumoral androgens in metastatic prostate cancer: a mechanism for castration-resistant tumor growth. Cancer Res.

[13] Pezaro CJ, Mukherji D, De Bono JS (2012). Abiraterone acetate: redefining hormone treatment for advanced prostate cancer. Drug Discov Today.

[14] Thakur A, Roy A, Ghosh A, Chhabra M, Banerjee S (2018). Abiraterone acetate in the treatment of prostate cancer. Biomed Pharmacother.

[15] Kallen J, Schlaeppi JM, Bitsch F, Filipuzzi I, Schilb A, Riou V (2004). Evidence for ligand-independent transcriptional activation of the human estrogen-related receptor alpha (ERRalpha): crystal structure of ERRalpha ligand binding domain in complex with peroxisome proliferator-activated receptor coactivator-1alpha. J Biol Chem.

[16] Deblois G, St-Pierre J, Giguere V (2013). The PGC-1/ERR signaling axis in cancer. Oncogene.

[17] Bianco S, Sailland J, Vanacker JM (2012). ERRs and cancers: effects on metabolism and on proliferation and migration capacities. J Steroid Biochem Mol Biol.

[18] Xu Z, Wang Y, Xiao ZG, Zou C, Zhang X, Wang Z (2018). Nuclear receptor ERRalpha and transcription factor ERG form a reciprocal loop in the regulation of TMPRSS2:ERG fusion gene in prostate cancer. Oncogene.

[19] Cheung CP, Yu S, Wong KB, Chan LW, Lai FM, Wang X (2005). Expression and functional study of estrogen receptor-related receptors in human prostatic cells and tissues. J Clin Endocrinol Metab.

[20] Fujimura T, Takahashi S, Urano T, Kumagai J, Ogushi T, Horie-Inoue K (2007). Increased expression of estrogen-related receptor alpha (ERRalpha) is a negative prognostic predictor in human prostate cancer. Int J Cancer.

[21] Zou C, Yu S, Xu Z, Wu D, Ng CF, Yao X (2014). ERRalpha augments HIF-1 signalling by directly interacting with HIF-1alpha in normoxic and hypoxic prostate cancer cells. J Pathol.

[22] Fradet A, Bouchet M, Delliaux C, Gervais M, Kan C, Benetollo C (2016). Estrogen related receptor alpha in castration-resistant prostate cancer cells promotes tumor progression in bone. Oncotarget.

[23] Grasso CS, Wu YM, Robinson DR, Cao X, Dhanasekaran SM, Khan AP (2012). The mutational landscape of lethal castration-resistant prostate cancer. Nature.

[24] Cai C, Wang H, He HH, Chen S, He L, Ma F (2013). ERG induces androgen receptor-mediated regulation of SOX9 in prostate cancer. J Clin Invest.

[25] Yu S, Wang MW, Yao X, Chan FL (2009). Establishment of a novel immortalized human prostatic epithelial cell line stably expressing androgen receptor and its application for the functional screening of androgen receptor modulators. Biochem Biophys Res Commun.

[26] Yu S, Wang X, Ng CF, Chen S, Chan FL (2007). ERRgamma suppresses cell proliferation and tumor growth of androgen-sensitive and androgen-insensitive prostate cancer cells and its implication as a therapeutic target for prostate cancer. Cancer Res.

[27] Xiao L, Wang Y, Xu K, Hu H, Xu Z, Wu D (2018). Nuclear Receptor LRH-1 Functions to Promote Castration-Resistant Growth of Prostate Cancer via Its Promotion of Intratumoral Androgen Biosynthesis. Cancer Res.

[28] Yu S, Wong YC, Wang XH, Ling MT, Ng CF, Chen S (2008). Orphan nuclear receptor estrogen-related receptor-beta suppresses in vitro and in vivo growth of prostate cancer cells via p21(WAF1/CIP1) induction and as a potential therapeutic target in prostate cancer. Oncogene.

[29] Vargas G, Bouchet M, Bouazza L, Reboul P, Boyault C, Gervais M (2019). ERRalpha promotes breast cancer cell dissemination to bone by increasing RANK expression in primary breast tumors. Oncogene.

[30] Yu S, Jia L, Zhang Y, Wu D, Xu Z, Ng CF (2013). Increased expression of activated endothelial nitric oxide synthase contributes to antiandrogen resistance in prostate cancer cells by suppressing androgen receptor transactivation. Cancer Lett.

[31] Yu S, Xu Z, Zou C, Wu D, Wang Y, Yao X (2014). Ion channel TRPM8 promotes hypoxic growth of prostate cancer cells via an O2 -independent and RACK1-mediated mechanism of HIF-1alpha stabilization. J Pathol.

[32] Patch RJ, Searle LL, Kim AJ, De D, Zhu X, Askari HB (2011). Identification of diaryl ether-based ligands for estrogen-related receptor alpha as potential antidiabetic agents. J Med Chem.

[33] Busch BB, Stevens WC Jr, Martin R, Ordentlich P, Zhou S, Sapp DW (2004). Identification of a selective inverse agonist for the orphan nuclear receptor estrogen-related receptor alpha. J Med Chem.

[34] Gordon JA, Noble JW, Midha A, Derakhshan F, Wang G, Adomat HH (2019). Upregulation of Scavenger Receptor B1 Is Required for Steroidogenic and Nonsteroidogenic Cholesterol Metabolism in Prostate Cancer. Cancer Res.

[35] Mitsiades N, Sung CC, Schultz N, Danila DC, He B, Eedunuri VK (2012). Distinct patterns of dysregulated expression of enzymes involved in androgen synthesis and metabolism in metastatic prostate cancer tumors. Cancer Res.

[36] Powell K, Semaan L, Conley-LaComb MK, Asangani I, Wu YM, Ginsburg KB (2015). ERG/AKR1C3/AR Constitutes a Feed-Forward Loop for AR Signaling in Prostate Cancer Cells. Clin Cancer Res.

[37] de Bono JS, Logothetis CJ, Molina A, Fizazi K, North S, Chu L (2011). Abiraterone and increased survival in metastatic prostate cancer. N Engl J Med.

[38] Fizazi K, Tran N, Fein L, Matsubara N, Rodriguez-Antolin A, Alekseev BY (2017). Abiraterone plus Prednisone in Metastatic, Castration-Sensitive Prostate Cancer. N Engl J Med.

[39] James ND, de Bono JS, Spears MR, Clarke NW, Mason MD, Dearnaley DP (2017). Abiraterone for Prostate Cancer Not Previously Treated with Hormone Therapy. N Engl J Med.

[40] Giacinti S, Bassanelli M, Aschelter AM, Milano A, Roberto M, Marchetti P (2014). Resistance to abiraterone in castration-resistant prostate cancer: a review of the literature. Anticancer Res.

[41] Mostaghel EA, Marck BT, Plymate SR, Vessella RL, Balk S, Matsumoto AM (2011). Resistance to CYP17A1 inhibition with abiraterone in castration-resistant prostate cancer: induction of steroidogenesis and androgen receptor splice variants. Clin Cancer Res.

[42] Chang KH, Li R, Kuri B, Lotan Y, Roehrborn CG, Liu J (2013). A gain-of-function mutation in DHT synthesis in castration-resistant prostate cancer. Cell.

[43] Tiwari A, Shivananda S, Gopinath KS, Kumar A (2014). MicroRNA-125a reduces proliferation and invasion of oral squamous cell carcinoma cells by targeting estrogen-related receptor alpha: implications for cancer therapeutics. J Biol Chem.

[44] Tiwari A, Swamy S, Gopinath KS, Kumar A (2015). Genomic amplification upregulates estrogen-related receptor alpha and its depletion inhibits oral squamous cell carcinoma tumors in vivo. Sci Rep.

[45] Paris PL, Andaya A, Fridlyand J, Jain AN, Weinberg V, Kowbel D (2004). Whole genome scanning identifies genotypes associated with recurrence and metastasis in prostate tumors. Hum Mol Genet.

