# Supplementary material for: Nuclear receptor ERRα contributes to castration-resistant growth of prostate cancer via its regulation of intratumoral androgen biosynthesis
Source: Theranostics. 2020 Mar 4;10(9):4201–16. doi: 10.7150/thno.35589 (PMC7086365; doi:10.7150/thno.35589)
Supplement: Supplementary file 1 — Supplementary figures and tables. [file thnov10p4201s1.pdf]

Tables S1

Primer pairs used for reporter gene cloning:

	Forward (5'-3')	Reverse (5'-3')
pGL3-AKR1C1-P1	CGGGGTACCAGGGTGAGTCC TCCAAGTGA	CCGCTCGAGCCCTCTGCAC ACTGCCTAAT
pGL3-AKR1C1-P3	CGGGGTACCGGCTAAAGCAT CAGCTCTGG	CCGCTCGAGTGTCAAACCT TTTTGCCACA
pGL3-AKR1C1-P1-deletion	CGGGGTACCAGGGTGAGTCC TCCAAGTGATTGATGGCAAG TCATGTGAT	CCGCTCGAGCCCTCTGCAC ACTGCCTAAT
pGL3-AKR1C1-P3- deletion	CGGGGTACCCCAGGAAGTG AGTTTCAGGAAATTTTACAT TAGGGTAATG	CATTACCCTAATGTAAAAT TTCCTGAAACTCACTTCCT GG
pGL3-CYP11A1	CGGGGTACCGTCAACTCTGT CCCGTATT	CCGCTCGAGCAGGTTTAGC CAGCCATT
pGL3-CYP11A1-P7-deletion	CGGGGTACCATACCCTGAGC TGTGCATGT	CCGCTCGAGCAGGTTTAGC CAGCCATT

Table S2

Primer pairs used for SYBR Green-based qRT-PCR assay:

Gene targets	Forward (5'-3')	Reverse (5'-3')
ACTB (β-actin)	ATGGATGATGATATCGCCGCG	CTCCATGTCGTCCCAGTTGGT
ESRRA (ERRα)	GTCCAAAGGGTTCCTCGGAG	GGATGCCACACCATAGTGGTA
AKR1C2	GTAAAGCTCTAGAGGCCGT	CACCCATGGTTCTTCTCGA
AKR1C3	GTAAAGCTTTGGAGGTCAC	CACCCATCGTTTGTCT
STAR	TACGTGGCTACTCAGCATCGAC	TCAACACCTGGCTTCAGAGGCA
HSD3B2	GGCTGTGCTGGCGGCTAAT	TGGAATCAAGGCGGAGGC
HSD17B3	TGAACGCACCGGATGAAATC	CTTGCAGGGCCTTGGAAAAT
CYP11A1	ACCAGAGACCCATAGGAGTCC	CAACAGGGGCAAAAAGTTCTTG
CYP17A1	GGCCCCATCTATTCGGTTCGT	GCGATACCCTTACGGTTGTTG
SRD5A2	CATACGGTTTAGCTTGGGTGT	GCTTTCCGAGATTTGGGGTAG
KLK3 (PSA)	TTGTCTTCCTCACCCTGTCC	TCACGCTTTTGTTCCTGATG
SR-B1	TCCTCACTTCCTCAACGCTG	TCCCAGTTTGTCCAATGCC

Table S3

Primer pairs used for ChIP-PCR assay:

	Forward (5’-3’)	Reverse (5’-3’)
AKR1C3-P1	CGTTGGAGCACCAGATGTAA	TGTATGCTGCCTTCCCTCTC
AKR1C3-P2	TTGAGCCAATTTTCCCAGAC	ACATTGTACTGGCCCAGAGG
AKR1C3-P3	GGCCATTAGAGGAAGCATCA	GGCCCATTTCCCTGGTATAA
AKR1C3-P4	ACCAGAGGCTGTCCTTGAGA	TCTGGGTTACATGCCAAACA
AKR1C3-P5	TGGGTACCACCCTCTCAGAC	AGAGGGAATGGGTGTCTCCT
CYP11A1-P1	TCTGGTATGGCCTTGAGC	AGGTCTGGCAGCCTTTGA
CYP11A1-P2	CTTATCCCTGTTGTTCAATG	GAAATATCTGCCTGTGCTT
CYP11A1-P3	GGGGAAAGCAGCACAGAT	TCACCTTGGAACCCTAAT
CYP11A1-P4	TCATTCCAGGCTCAAGGT	GCCCATAGATCACCCACA
CYP11A1-P5	GTGCTGAGACCCTTGGAG	ATGACCTTGAGCCTGGAA
CYP11A1-P6	TTGGCTGTACCTGCTGTG	AGAAGGCGGAAGAACTCA
CYP11A1-P7	AATCCCTGACTACATAAGC	AGATGGGACTCAAGGTTAT
Known ERRE	CCATCCGAGTGGAATTTGAG TCCTAAAG	GAACCGTAGACCCAGTAGCCCC ACAGAG
Negative control	GGACTACCAAGGAGAAGCTA	ATTAATAGCCCTGCCTGGAT

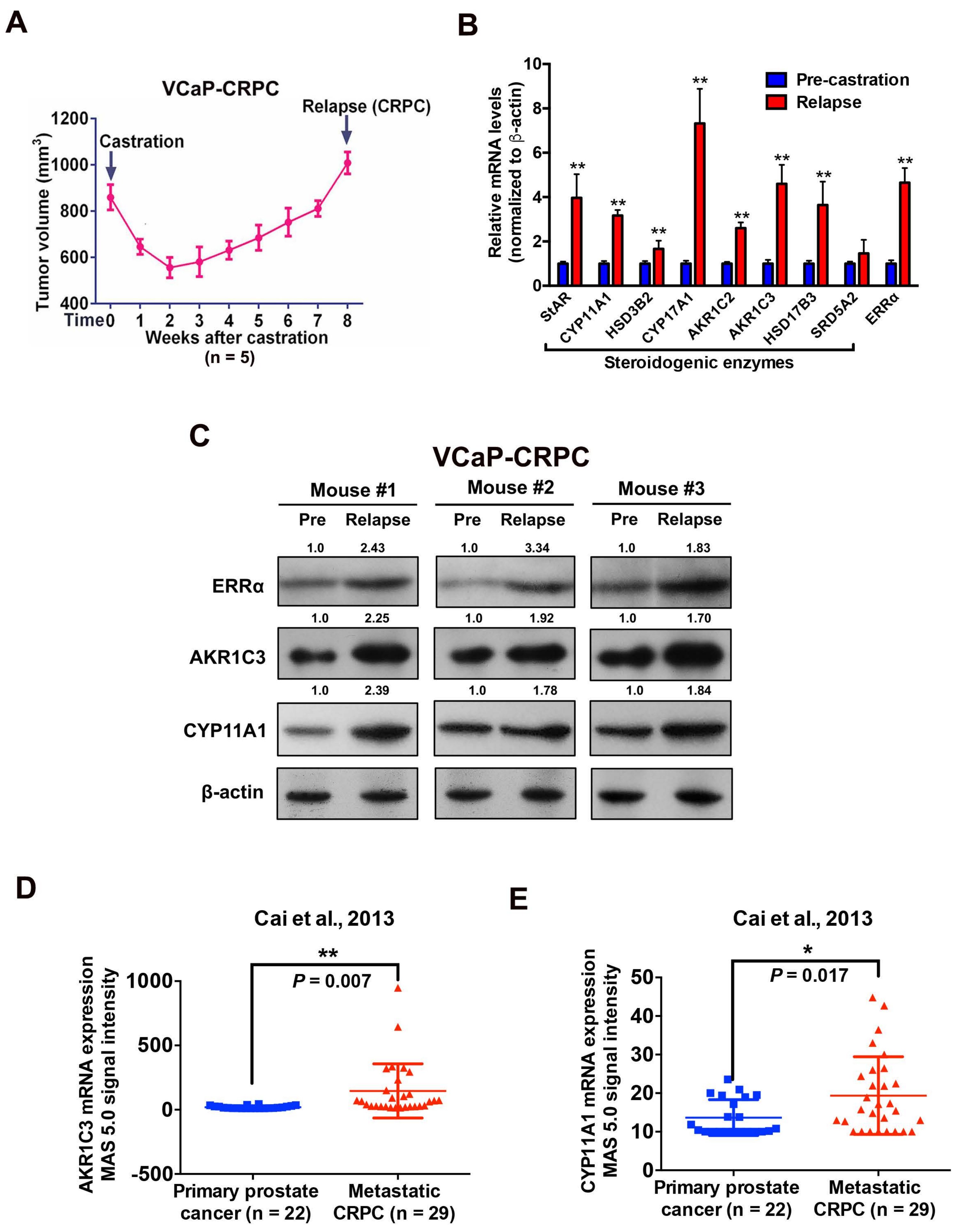
A
VCaP-CRPC
Relapse (CRPC)
Castration
Tumor volume (mm3)
1200
1000
800
600
400
Time 0 1 2 3 4 5 6 7 8
Weeks after castration
(n = 5)
B
Relative mRNA levels
(normalized to β-actin)
Pre-castration
Relapse
StAR
CYP11A1
HSD3B2
CYP17A1
AKR1C2
AKR1C3
HSD17B3
SRD5A2
ERRα
Steroidogenic enzymes
C
VCaP-CRPC
Mouse #1
Mouse #2
Mouse #3
Pre Relapse
1.0 2.43
1.0 3.34
1.0 1.83
ERRα
1.0 2.25
1.0 1.92
1.0 1.70
AKR1C3
1.0 2.39
1.0 1.78
1.0 1.84
CYP11A1
β-actin
D
Cai et al., 2013
AKR1C3 mRNA expression
MAS 5.0 signal intensity
P = 0.007
Primary prostate cancer (n = 22)
Metastatic CRPC (n = 29)
E
Cai et al., 2013
CYP11A1 mRNA expression
MAS 5.0 signal intensity
P = 0.017
Primary prostate cancer (n = 22)
Metastatic CRPC (n = 29)

Figure S1

Figure S1. (A-C) VCaP-CRPC castration-relapse prostate cancer xenograft tumors exhibit increased expression of ERRα and numerous steroidogenic enzymes. **(A)** VCaP-CRPC xenograft tumor model was established based on the relapse of tumors in host mice after castration. **(B)** qRT-PCR analysis. Castration-relapse VCaP-CRPC xenograft tumors expressed significant higher transcript levels of ERRα and a panel of key steroidogenic enzymes (including, *STAR, CYP11A1, HSD3B2, CYP17A1, AKR1C2, AKR1C3, HSD17B3* and *SDR5A2*) as compared to same tumors before castration. **(C)** Immunoblot analysis. VCaP-CRPC tumors expressed higher protein levels of ERRα, AKR1C3 and CYP11A1 as compared to pre-castration (Pre) VCaP tumors. Data are represented as mean ± SD (n = 3) and analyzed by Students' *t*-test. **, $P < 0.01$ versus pre-castration VCaP tumors. **(D and E)** mRNA Expression profiles of **(D)** AKR1C3 and **(E)** CYP11A1 as revealed from a GEO cohort (GSE32269; Cai et al., 2013) of metastatic CRPC. Results showed that bone-metastatic CRPC samples exhibited significant higher mRNA levels of AKR1C3 and CYP11A1 as compared to localized prostate cancer.

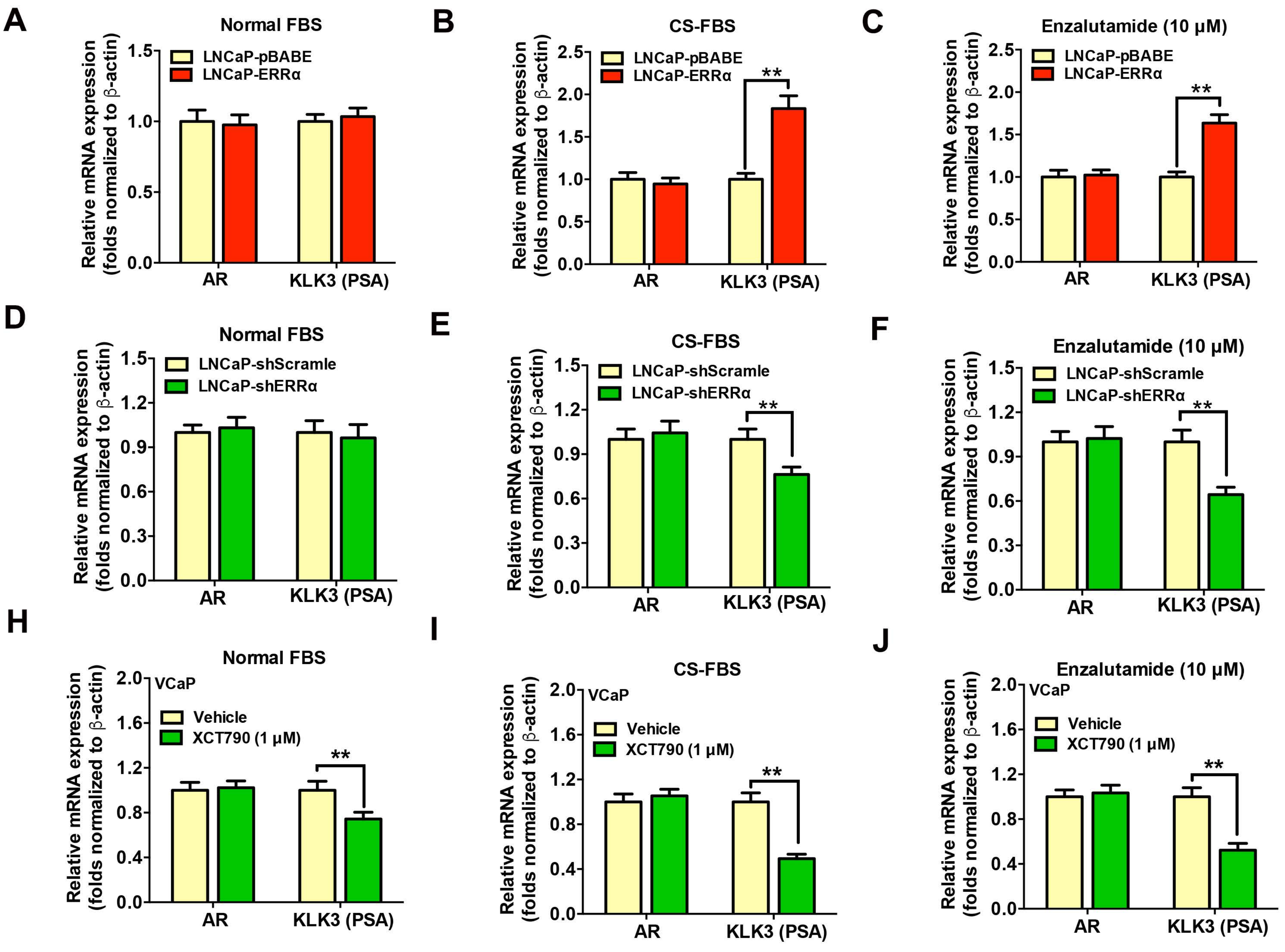
A
Normal FBS
LNCaP-pBABE
LNCaP-ERRα
Relative mRNA expression (folds normalized to β-actin)
AR
KLK3 (PSA)
B
CS-FBS
LNCaP-pBABE
LNCaP-ERRα
**
Relative mRNA expression (folds normalized to β-actin)
AR
KLK3 (PSA)
C
Enzalutamide (10 μM)
LNCaP-pBABE
LNCaP-ERRα
**
Relative mRNA expression (folds normalized to β-actin)
AR
KLK3 (PSA)
D
Normal FBS
LNCaP-shScramle
LNCaP-shERRα
Relative mRNA expression (folds normalized to β-actin)
AR
KLK3 (PSA)
E
CS-FBS
LNCaP-shScramle
LNCaP-shERRα
**
Relative mRNA expression (folds normalized to β-actin)
AR
KLK3 (PSA)
F
Enzalutamide (10 μM)
LNCaP-shScramle
LNCaP-shERRα
**
Relative mRNA expression (folds normalized to β-actin)
AR
KLK3 (PSA)
H
Normal FBS
VCaP
Vehicle
XCT790 (1 μM)
**
Relative mRNA expression (folds normalized to β-actin)
AR
KLK3 (PSA)
I
CS-FBS
VCaP
Vehicle
XCT790 (1 μM)
**
Relative mRNA expression (folds normalized to β-actin)
AR
KLK3 (PSA)
J
Enzalutamide (10 μM)
VCaP
Vehicle
XCT790 (1 μM)
**
Relative mRNA expression (folds normalized to β-actin)
AR
KLK3 (PSA)

Figure S2

Figure S2. qPCR analyses of AR and PSA mRNA levels in experiments conducted in Figure 2. **(A-C)** qRT-PCR analysis of AR and PSA mRNA expression levels in LNCaP-ERRα and LNCaP-pBABE cells under normal culture condition, androgen-deprivation culture condition (CS-FBS) and Enzalutamide treatment. **(A)** Results showed that AR and PSA mRNA expression levels showed no difference in LNCaP-ERRα as compared to LNCaP-pBABE cells under normal culture condition. **(C and D)** However, when being cultured with CS-FBS or Enzalutamide, LNCaP-ERRα cells exhibited increased expression level of PSA mRNA as compared to LNCaP-pBABE cells. **(D-F)** qRT-PCR analysis of AR and PSA mRNA expression levels in LNCaP-shERRα and LNCaP-shScramble cells under normal culture condition, androgen-deprivation culture condition and Enzalutamide treatment. **(D)** Results showed that AR and PSA mRNA expression levels showed no difference in LNCaP-shERRα as compared to LNCaP-shScramble cells under normal culture condition. **(E and F)** However, when being cultured with CS-FBS or Enzalutamide, LNCaP-shERRα cells exhibited decreased expression level of PSA mRNA as compared to LNCaP- shScramble cells. **(H-J)** XCT790 treatment of VCaP cells. XCT790 treatment could significantly suppress the expression of PSA mRNA in VCaP cells cultured with normal FBS, with more significant inhibitory effect under androgen-deprivation culture condition or Enzalutamide treatment.

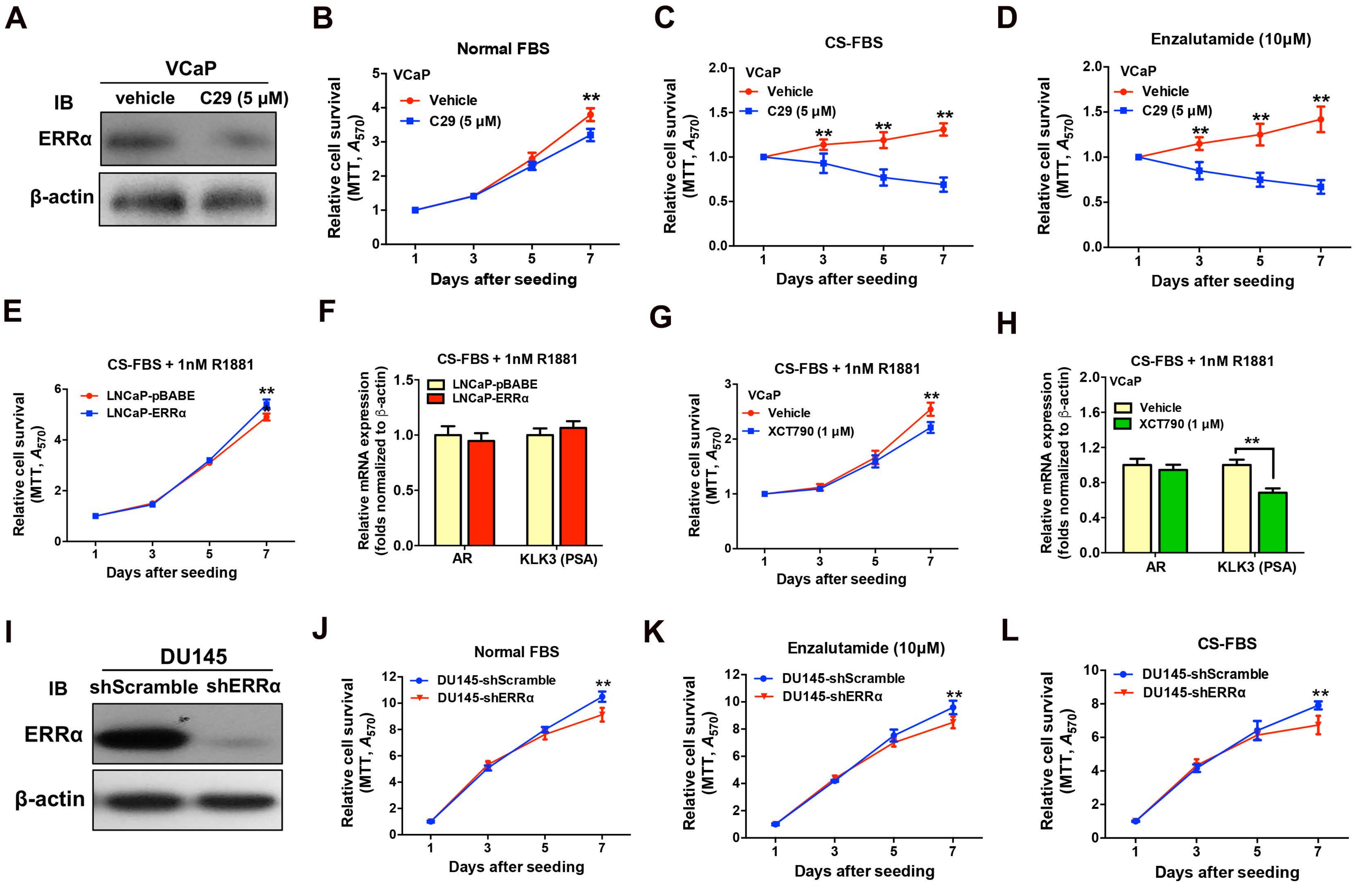
A
VCaP
IB
vehicle
C29 (5 μM)
ERRα
β-actin
B
Normal FBS
VCaP
Vehicle
C29 (5 μM)
**
Relative cell survival (MTT, A570)
Days after seeding
C
CS-FBS
VCaP
Vehicle
C29 (5 μM)
D
Enzalutamide (10μM)
VCaP
Vehicle
C29 (5 μM)
E
CS-FBS + 1nM R1881
LNCaP-pBABE
LNCaP-ERRα
F
CS-FBS + 1nM R1881
LNCaP-pBABE
LNCaP-ERRα
Relative mRNA expression (folds normalized to β-actin)
AR
KLK3 (PSA)
G
CS-FBS + 1nM R1881
VCaP
Vehicle
XCT790 (1 μM)
H
CS-FBS + 1nM R1881
VCaP
Vehicle
XCT790 (1 μM)
I
DU145
IB
shScramble
shERRα
ERRα
β-actin
J
Normal FBS
DU145-shScramble
DU145-shERRα
K
Enzalutamide (10μM)
DU145-shScramble
DU145-shERRα
L
CS-FBS
DU145-shScramble
DU145-shERRα

Figure S3

Figure S3. (A) Immunoblot analysis showed that C29 treatment could significantly reduce or abolish the protein level of ERRα in VCaP cells. **(B-D)** C29 treatment could significantly suppress the *in vitro* growth of VCaP cells cultured with CS-FBS or Enzalutamide. **(E)** *In vitro* growth responses of LNCaP-pBABE and LNCaP-ERRα cells cultured in CS-FBS supplemented with R1881 assayed by MTT. The suppressed growth of LNCaP-ERRα and LNCaP-pBABE cells cultured with CS-FBS could be restored upon supplement with R1881, with no significant difference on their growth responses between the two treated groups until Day-7. **(F)** qRT-PCR analysis of AR and PSA mRNA expression levels in LNCaP-ERRα and LNCaP-pBABE cells cultured in CS-FBS supplemented with R1881 for 7 days. Results showed that AR and PSA mRNA expression levels showed no difference in LNCaP-ERRα as compared to LNCaP-pBABE cells. **(G)** *In vitro* growth responses of VCaP cell upon XCT790 or vehicle treatment cultured with CS-FBS supplemented with R1881 assayed by MTT. The suppressed growth of VCaP cultured with CS-FBS could be restored upon supplement with R1881, with no significant difference on their growth responses upon XCT790 or vehicle treatment until day-7. **(H)** qRT-PCR analysis of AR and PSA mRNA in VCaP cells cultured with CS-FBS supplemented with R1881 for 7 days. Results showed that PSA mRNA expression levels was reduced in VCaP cell upon XCT790 treatment as compared to vehicle without change of AR mRNA levels. **(I)** Immunoblot validation of stable ERRα-knockdown in DU145-shERRα cells. **(J-L)** *In vitro* growth responses of DU145-shERRα cells cultured with normal FBS, CS-FBS or Enzalutamide. No significant change in growth responses was observed in DU145-shERRα cells upon cultures with normal FBS **(J)**, charcoal-stripped FBS **(K)** and Enzalutamide **(L)**, as compared to DU145-shScramble cells until Day-7. **, $P < 0.01$ versus DU145-shScramble cells.

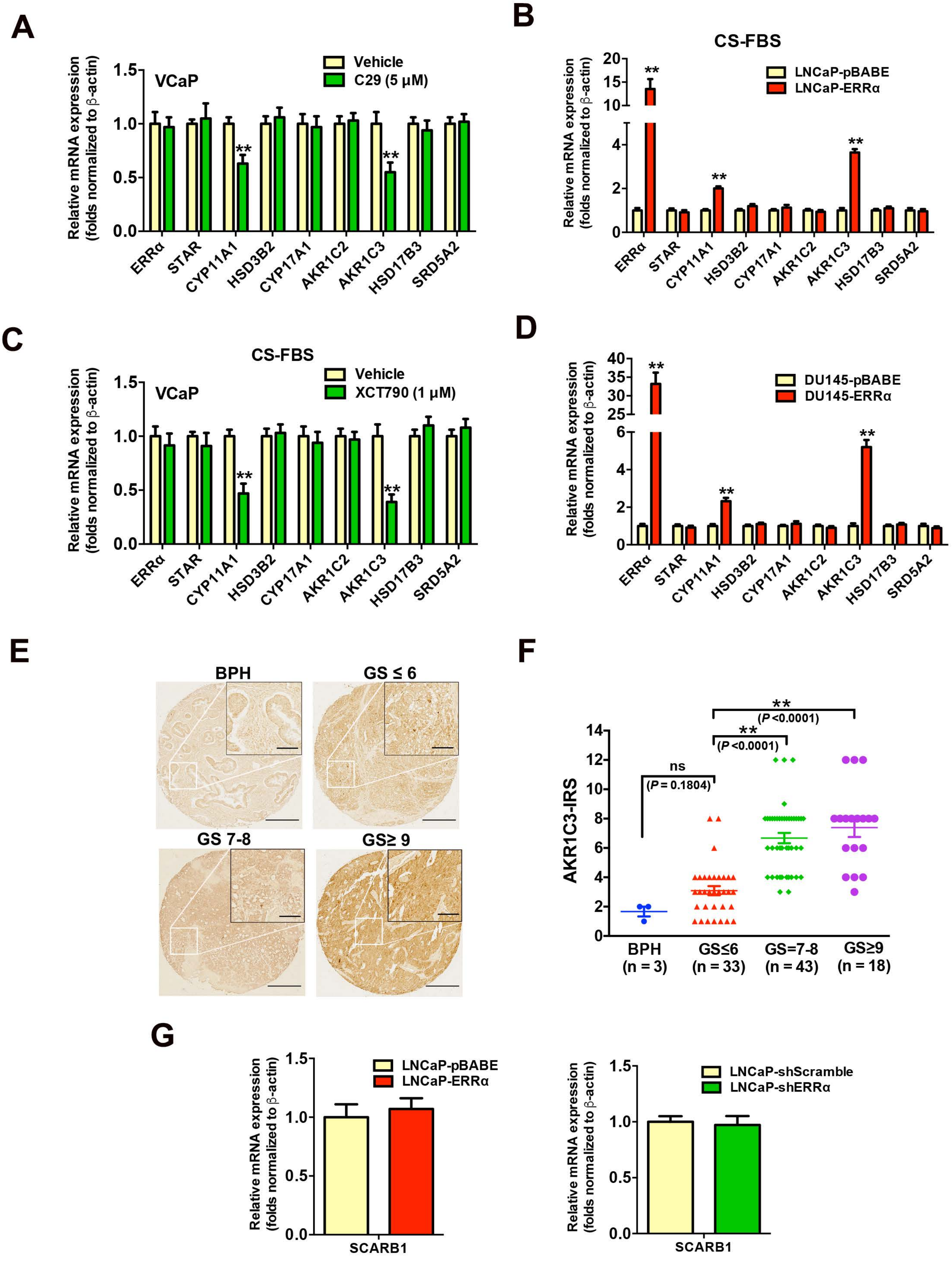

A
VCaP
Vehicle
C29 (5 μM)
Relative mRNA expression (folds normalized to β-actin)
1.5
1.0
0.5
0.0
**
**
ERRα
STAR
CYP11A1
HSD3B2
CYP17A1
AKR1C2
AKR1C3
HSD17B3
SRD5A2
B
CS-FBS
LNCaP-pBABE
LNCaP-ERRα
Relative mRNA expression (folds normalized to β-actin)
20
15
10
4
2
0
**
**
**
ERRα
STAR
CYP11A1
HSD3B2
CYP17A1
AKR1C2
AKR1C3
HSD17B3
SRD5A2
C
CS-FBS
VCaP
Vehicle
XCT790 (1 μM)
Relative mRNA expression (folds normalized to β-actin)
1.5
1.0
0.5
0.0
**
**
ERRα
STAR
CYP11A1
HSD3B2
CYP17A1
AKR1C2
AKR1C3
HSD17B3
SRD5A2
D
DU145-pBABE
DU145-ERRα
Relative mRNA expression (folds normalized to β-actin)
40
35
30
25
6
4
2
0
**
**
**
ERRα
STAR
CYP11A1
HSD3B2
CYP17A1
AKR1C2
AKR1C3
HSD17B3
SRD5A2
E
BPH
GS ≤ 6
GS 7-8
GS≥ 9
F
AKR1C3-IRS
14
12
10
8
6
4
2
0
ns
(P = 0.1804)
**
(P <0.0001)
**
(P <0.0001)
BPH (n = 3)
GS≤6 (n = 33)
GS=7-8 (n = 43)
GS≥9 (n = 18)
G
LNCaP-pBABE
LNCaP-ERRα
Relative mRNA expression (folds normalized to β-actin)
1.5
1.0
0.5
0.0
SCARB1
LNCaP-shScramble
LNCaP-shERRα
Relative mRNA expression (folds normalized to β-actin)
1.5
1.0
0.5
0.0
SCARB1

Figure S4

Figure S4. (A-D) qRT-PCR analysis. **(A)** C29 treatment. C29 treatment could significantly suppress the mRNA levels of CYP11A1 and AKR1C3 in VCaP cells. **(B)** LNCaP-ERRα cells expressed higher transcript levels of CYP11A1 and AKR1C3 as compared to empty vector LNCaP-pBABE cells under androgen-deprivation culture condition (CS-FBS). **(C)** XCT790 treatment. XCT790 treatment could significantly suppress the mRNA levels of CYP11A1 and AKR1C3 in VCaP cells under androgen-deprivation culture condition (CS-FBS). **(D)** DU145-ERRα cells expressed higher transcript levels of CYP11A1 and AKR1C3 as compared to empty vector DU145-pBABE cells. **(E)** AKR1C3 immunohistochemistry. Representative micrographs of AKR1C3-immunostained tissue microarray spots of benign prostatic hyperplasia (BPH) and malignant lesions. A significant increase of cytoplasmic AKR1C3 immunosignals was detected in malignant cells in adenocarcinoma lesions with higher Gleason scores. Magnification, ×40; scale bars, 250 μm. Insets show the enclosed areas at higher magnification. Magnification, ×200; scale bars, 50 μm. **(F)** AKR1C3 immunoreactive score (AKR1C3-IRS) performed on BPH and neoplastic prostatic tissues. Adenocarcinomas with higher Gleason scores (GS $\geq$ 7) showed significantly higher AKR1C3 expression than benign hyperplastic tissues. **(G)** qRT-PCR analysis. SCARB1 mRNA expression levels showed no difference in either LNCaP-ERRα or LNCaP-shERRα cells as compared to their control LNCaP-pBABE or LNCaP shScramble cells. **, $P < 0.01$ versus LNCaP-pBABE cells, LNCaP-shScramble cells, DU145-shScramble cells or vehicle treatment.

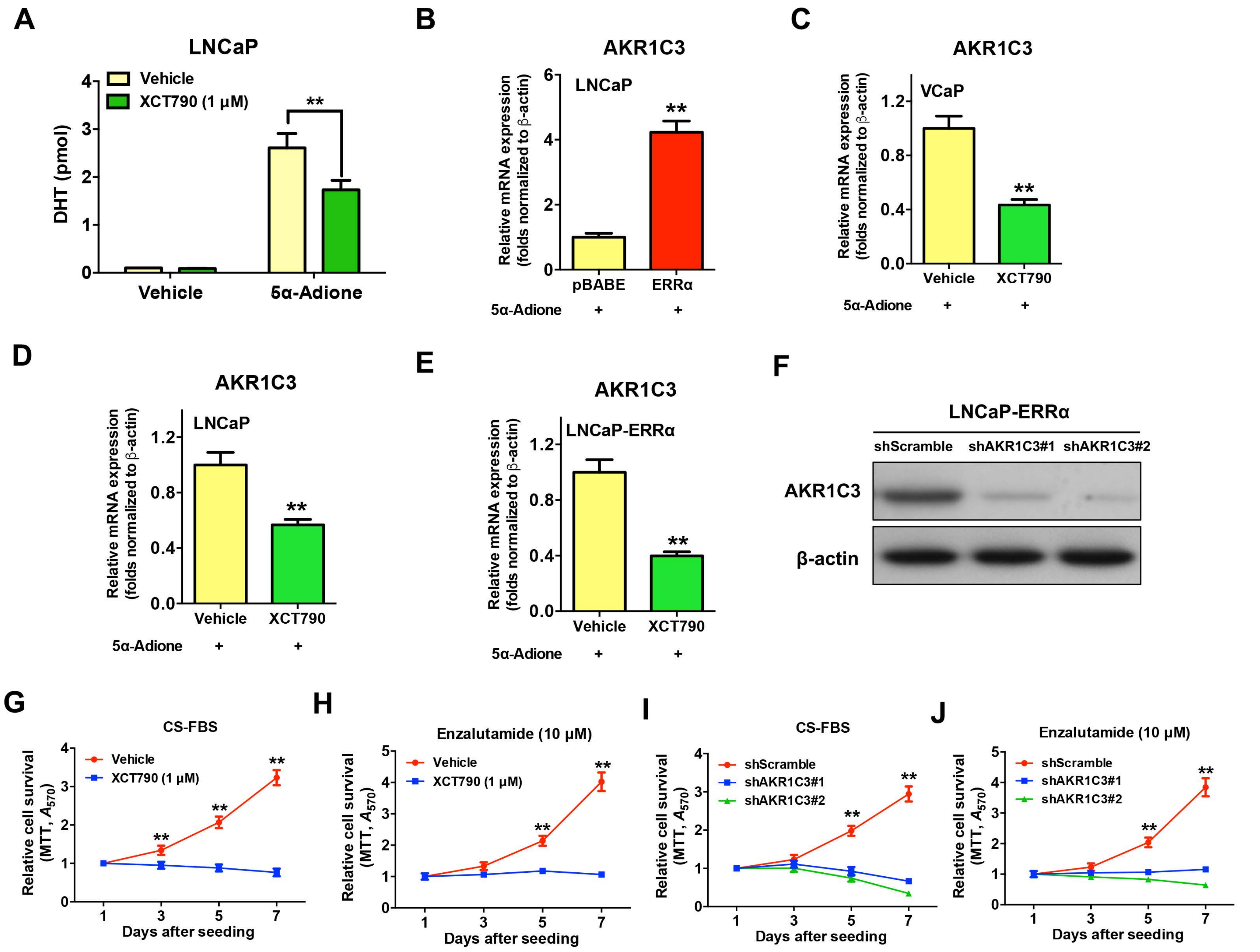

A
LNCaP
Vehicle
XCT790 (1 μM)
DHT (pmol)
Vehicle
5α-Adione
B
AKR1C3
LNCaP
Relative mRNA expression (folds normalized to β-actin)
pBABE
ERRα
5α-Adione
C
AKR1C3
VCaP
Vehicle
XCT790
D
AKR1C3
LNCaP
E
AKR1C3
LNCaP-ERRα
F
LNCaP-ERRα
shScramble
shAKR1C3#1
shAKR1C3#2
AKR1C3
β-actin
G
CS-FBS
Relative cell survival (MTT, A_{570})
Days after seeding
H
Enzalutamide (10 μM)
I
CS-FBS
shScramble
shAKR1C3#1
shAKR1C3#2
J
Enzalutamide (10 μM)

Figure S5

Figure S5. (A) UPLC-MS/MS measurement of DHT in XCT790-treated LNCaP cells. Results showed that suppression of ERRα activity by XCT790 could significantly reduce the DHT level in 5α-Adione-supplemented LNCaP cells. **(B-E)** qPCR analyses of AKR1C3 mRNA levels in experiments conducted in Figures 6B-D and Figure 4SA. **(F)** Immunoblot validation of stable AKR1C3-knockdown in LNCaP-ERRα cells. **(G-J)** XCT790 treatment and AKR1C3 knockdown could significantly could re-sensitize LNCaP-ERRα cells to culture condition with CS-FBS or Enzalutamide.

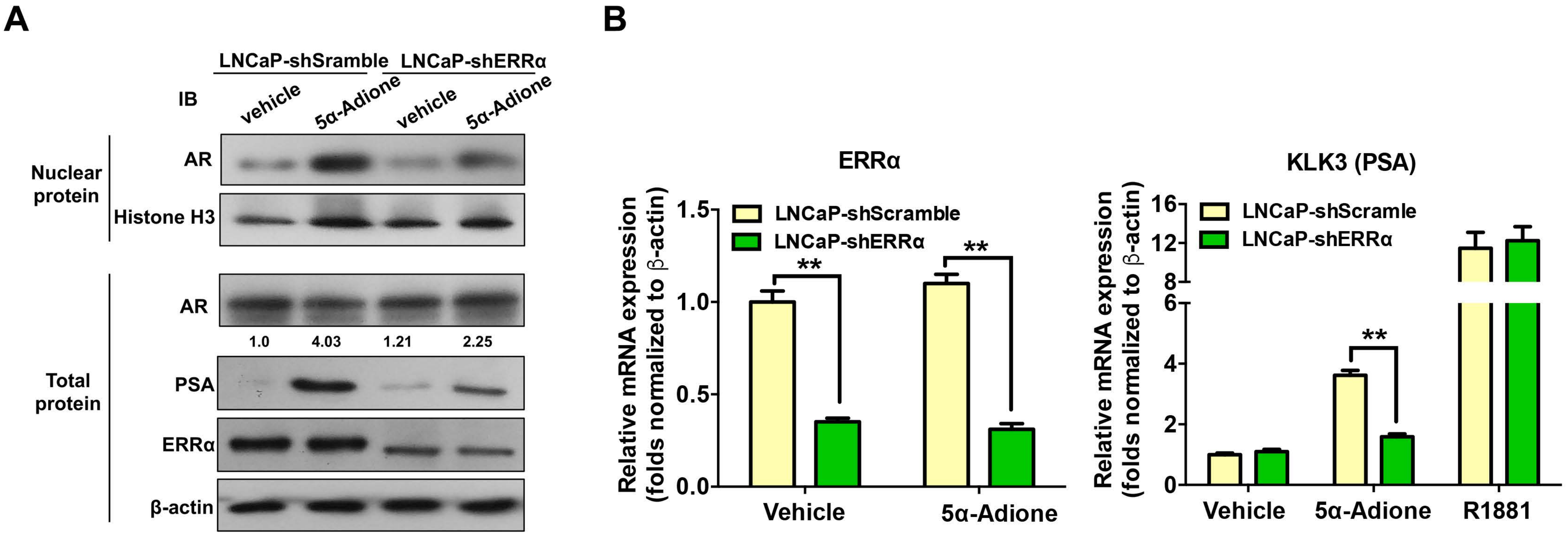
A
LNCaP-shSramble
LNCaP-shERRα
IB
vehicle
5α-Adione
vehicle
5α-Adione
Nuclear protein
AR
Histone H3
Total protein
AR
1.0
4.03
1.21
2.25
PSA
ERRα
β-actin
B
ERRα
Relative mRNA expression (folds normalized to β-actin)
1.5
1.0
0.5
0.0
LNCaP-shScramble
LNCaP-shERRα
**
**
Vehicle
5α-Adione
KLK3 (PSA)
Relative mRNA expression (folds normalized to β-actin)
16
12
8
6
4
2
0
LNCaP-shScramle
LNCaP-shERRα
**
Vehicle
5α-Adione
R1881

Figure S6

Figure S6. ERRα-knockdown attenuates the AR signaling in prostate cancer cells. **(A)** Immunoblot analysis of AR and PSA expressions in LNCaP-shERRα and LNCaP-shScramble transduced cells. Results showed that knockdown of ERRα could lessen the enhanced nuclear AR and PSA levels induced by 5α-Adione supplement. **(B)** qRT-PCR analysis of ERRα and KLK3 (PSA) expression in 5α-Adione-supplemented LNCaP-shERRα and LNCaP-shScramble cells. Knockdown of ERRα could significantly reduce the KLK3 mRNA level in LNCaP-shERRα cells as compared to LNCaP-shScramble cells, with 5α-Adione supplement. Supplement with R1881 induced increase of PSA mRNA levels in both LNCaP-shScramble and LNCaP-shERRα cells at same levels. **, $P < 0.01$ versus LNCaP-shScramble cells.

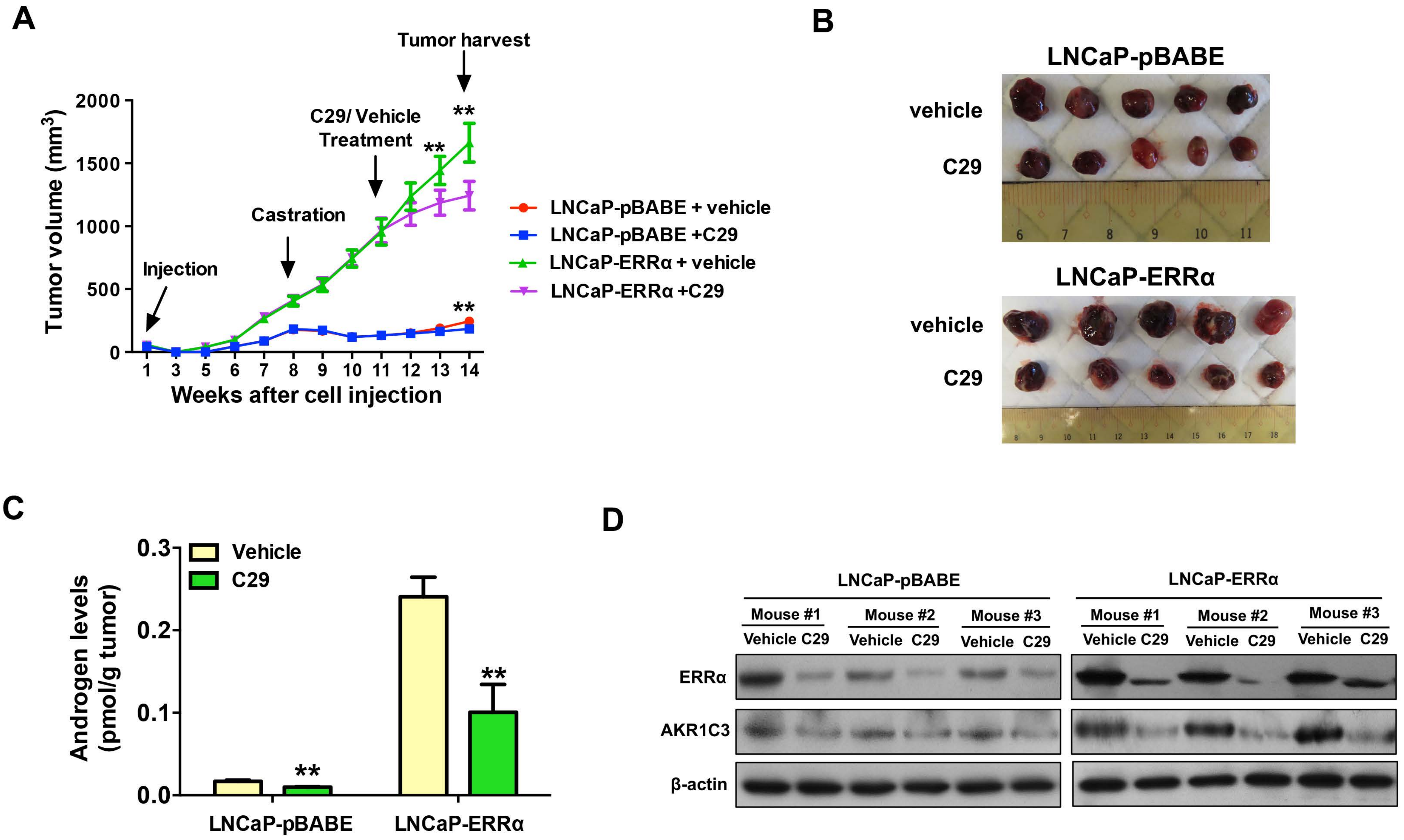
A
Tumor harvest
C29/ Vehicle
Treatment
Castration
Injection
**
**
**
Tumor volume (mm^3)
2000
1500
1000
500
0
1
3
5
6
7
8
9
10
11
12
13
14
Weeks after cell injection
LNCaP-pBABE + vehicle
LNCaP-pBABE +C29
LNCaP-ERRα + vehicle
LNCaP-ERRα +C29
B
LNCaP-pBABE
vehicle
C29
LNCaP-ERRα
vehicle
C29
C
Vehicle
C29
Androgen levels
(pmol/g tumor)
0.3
0.2
0.1
0.0
**
**
LNCaP-pBABE
LNCaP-ERRα
D
LNCaP-pBABE
LNCaP-ERRα
Mouse #1
Mouse #2
Mouse #3
Vehicle C29
ERRα
AKR1C3
β-actin

Figure S7

Figure S7. ERRα inverse agonist C29 suppresses *in vivo* castration-resistant growth of prostate cancer cells. **(A)** Growth curve of castration-relapsed LNCaP-pBABE and LNCaP-ERRα xenograft tumors upon C29 or vehicle treatment for 3 weeks. Results showed that C29 could significantly retard the castration-relapsed growth of both LNCaP-pBABE and LNCaP-ERRα tumors as compared to vehicle. **(B)** Images show the representative dissected LNCaP-pBABE and LNCaP-ERRα xenograft tumors upon 3-week treatment with C29 or vehicle in castrated host. **(C)** Measurement of DHT in LNCaP-pBABE and LNCaP-ERRα xenograft tumors by LC-MS/MS. Significant reduction of DHT levels was detected in tumors upon C29 treatment as compared to vehicle. **(D)** Immunoblot analysis. LNCaP-pBABE and LNCaP-ERRα tumors upon C29 treatment expressed lower protein levels of ERRα and AKR1C3 as compared to vehicle. **, $P < 0.01$ versus vehicle.
